# Neural speech tracking shifts from the syllabic to the modulation rate of speech as intelligibility decreases

**DOI:** 10.1111/psyp.14362

**Published:** 2023-06-23

**Authors:** Fabian Schmidt, Ya‐Ping Chen, Anne Keitel, Sebastian Rösch, Ronny Hannemann, Maja Serman, Anne Hauswald, Nathan Weisz

**Affiliations:** ^1^ Center for Cognitive Neuroscience University of Salzburg Salzburg Austria; ^2^ Department of Psychology University of Salzburg Salzburg Austria; ^3^ Psychology, School of Social Sciences University of Dundee Dundee UK; ^4^ Department of Otorhinolaryngology Paracelsus Medical University Salzburg Austria; ^5^ Audiological Research Unit Sivantos GmbH Erlangen Germany; ^6^ Neuroscience Institute, Christian Doppler University Hospital, Paracelsus Medical University Salzburg Austria

**Keywords:** MEG, neural speech tracking, spectral parametrization, speech envelope modulation, speech‐brain coherence, syllable rate

## Abstract

The most prominent acoustic features in speech are intensity modulations, represented by the amplitude envelope of speech. Synchronization of neural activity with these modulations supports speech comprehension. As the acoustic modulation of speech is related to the production of syllables, investigations of neural speech tracking commonly do not distinguish between lower‐level acoustic (envelope modulation) and higher‐level linguistic (syllable rate) information. Here we manipulated speech intelligibility using noise‐vocoded speech and investigated the spectral dynamics of neural speech processing, across two studies at cortical and subcortical levels of the auditory hierarchy, using magnetoencephalography. Overall, cortical regions mostly track the syllable rate, whereas subcortical regions track the acoustic envelope. Furthermore, with less intelligible speech, tracking of the modulation rate becomes more dominant. Our study highlights the importance of distinguishing between envelope modulation and syllable rate and provides novel possibilities to better understand differences between auditory processing and speech/language processing disorders.

## Abstract 2

Natural speech consists of both acoustic sound modulations and linguistic features, such as the rate at which syllables are expressed. Current investigations of neural speech tracking do not distinguish between low‐level acoustic and linguistic information. Instead, the low‐frequency acoustic modulations of speech are commonly simply equated to the production of syllables. However, while the temporal scale of the low‐level acoustic modulations of speech is surprisingly stable across various speech settings (different speakers, languages, and speaking conditions) the production rate of syllables can differ across the same settings. We show that cortical regions track the syllabic rate, whereas subcortical regions track the modulation rate of speech. Furthermore, with declining intelligibility tracking of the modulation rate becomes overall more dominant.

## 1 INTRODUCTION

Intensity modulations of the acoustic envelope reflect the most prominent feature of the acoustic speech stream. Synchronization of neural activity with these modulations supports speech comprehension (Doelling et al., 2014; Gross et al., 2013; Keitel et al., 2018; Peelle et al., 2013). As the acoustic modulation of speech and the production of syllables is correlated (Poeppel & Assaneo, 2020), investigations of neural speech tracking commonly do not distinguish between acoustic (envelope modulation) and related linguistic (syllable rate) information. However, while the temporal scale of the acoustic modulation (~4–5 Hz) is remarkably similar across languages, speakers, and speaking conditions (for reviews see (Ding et al., 2017; Poeppel & Assaneo, 2020)), the rate at which syllables are produced can vary significantly across (and within) languages (Coupé et al., 2019), dialects, and speaking conditions (Jacewicz et al., 2009). Therefore, it remains unclear whether and how the brain differentially tracks low‐level acoustic and linguistic information during natural continuous speech. Distinguishing these aspects more clearly may also be important in gaining a better understanding of the neural processes separating auditory processing disorders (e.g., hearing loss) from language processing disorders (e.g., developmental dyslexia).

The ability to process meaningful information from an acoustic sound stream becomes especially important in difficult listening situations. While some studies indicate a positive relationship between speech intelligibility and the synchronization of brain activity with the speech envelope (neural speech tracking) in the low‐frequency range (Doelling et al., 2014; Gross et al., 2013; Keitel et al., 2018; Peelle et al., 2013) others have reported inverse effects (Ding et al., 2014; Song & Iverson, 2018). A recent study even suggested an inverted u‐shaped relationship, where synchronization increases when speech is mildly degraded and decreases as speech becomes unintelligible (Hauswald et al., 2022). This wide range of (partly contradicting) results is suggestive of a complex relationship between the intelligibility of speech and the related neural dynamics of speech tracking.

One source of these seeming inconsistencies may be related to the interpretation of band‐limited differences, conflating periodic (center frequency, power, bandwidth) and aperiodic (offset, exponent) properties of the underlying signals (Donoghue et al., 2020). In fact, both the acoustic envelope of speech and electrophysiological measurements of neural activity possess an overall 1/f‐like spectrum (Pritchard, 1992; Voss & Clarke, 1975). This 1/f‐like pattern is also at times present in the low‐frequency coherence/correlation spectrum between both signals (e.g., see (Ding et al., 2014; Gross et al., 2013; Hauswald et al., 2022)). Recently, several approaches were proposed to separate periodic from aperiodic components of electrophysiological activity (*IRASA* (Wen & Liu, 2016); *FOOOF* (Donoghue et al., 2020)). We applied one of these approaches (*FOOOF*) to speech tracking, to parametrize the periodic components underlying low‐frequency speech‐brain coherence, such as the center frequency, the relative height of the coherence peak, and its bandwidth (~tuning). Commonly, when investigating neural speech tracking these parameters are not separated from the aperiodic components of the coherence spectra. Instead (band/averaged) contrasts over coherence spectra across several experimental conditions are computed, conflating the periodic and aperiodic components underlying speech‐brain coherence. We propose that the periodic components (center frequency, relative height of the coherence peak, bandwidth) of speech‐brain coherence offer a better estimate of neural speech tracking than broadband speech‐brain coherence in the conventional frequency ranges. Therefore, it may be beneficial to investigate these parameters separately to better understand how neural activity tracks acoustic and linguistic information in a continuous speech stream and how this tracking is influenced by speech intelligibility.

Here, we applied this approach to two separate studies in which speech intelligibility was parametrically controlled via vocoding (3‐, 7‐Channels or no vocoding). Vocoding (Shannon et al., 1995) is a popular technique to manipulate the intelligibility of speech that allows for high parametric control, while only moderately influencing the acoustic envelope of the signal (Peelle et al., 2013). We captured the spectral dynamics of neural speech processing at cortical and subcortical levels (Schmidt et al., 2020) of the auditory hierarchy using magnetoencephalography (MEG). We observed that low‐frequency speech‐brain coherence in accordance with previous results (Doelling et al., 2014; Gross et al., 2013; Keitel et al., 2018; Peelle et al., 2013) declines with a decrease in intelligibility. However, parametrization of the coherence spectra revealed that this effect was mainly driven by the aperiodic components. The periodic components that are presumably reflective of neural speech tracking (opposed to band‐limited coherence differences) were characterized by a narrower frequency tuning of the low‐frequency coherence peak of vocoded speech along with an increase in its center frequency. The latter effect points to a shift of cortical tracking away from the syllabic rate toward the general acoustic modulation rate of the speech envelope as vocoding increased. This effect is also seen for subcortical regions, although tracking is here overall dominated by the acoustic modulation rate.

## 2 RESULTS

### 2.1 Task performance declines with speech intelligibility

**FIGURE 1:**
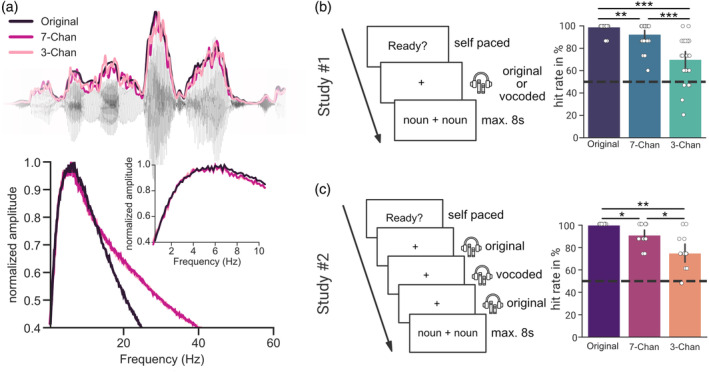
Task performance declines with speech intelligibility (a) An excerpt from the audiobook presented with the corresponding speech envelope (Original) and with the envelopes of the vocoded audio stimuli (7‐Channels and 3‐Channels) and the averaged modulation spectra of the audio streams. (b) In Study#1 subjects listened either to a continuous segment of clear or vocoded speech. (c) In Study#2 short segments of vocoded speech ~6–18 s were embedded in an otherwise clear speech stream (~1–3 min duration). In both studies, subjects were presented with two nouns at the end of each stimulus. They were further instructed to pick the one they perceived in the previous sentence. Hit rates declined in both experiments with a decrease in speech intelligibility. Chan, Channels. *p* < 0.05*, *p* < 0.01**, *p* < 0.001***.

Subjects (*N* = 55 across two experiments; Figure 1b,c) listened to an audiobook (“Das Märchen”; Goethe, 1795) narrated by a female speaker while seated in the MEG. Parts of the audiobook presented were noise‐vocoded (Figure 1a; 7‐Chan, 3‐Chan). Vocoding levels were either kept constant throughout the audio presentation (Study#1; Figure 1b) or changed intermittently (Study#2; Figure 1c) to test the influences of vocoded speech on neural speech tracking under two different conditions. At the end of each audio presentation, subjects were presented with two nouns from which they had to pick the one they perceived in the previous sentence. The audio presentations were embedded in blocks that varied between 3.5 and 9 min (see Section 5 (Method) for a detailed account). Due to the overall low number of behavioral responses, we added an additional behavioral assessment (adjusted for each study) to investigate how vocoding influences speech comprehension. The task was similar to the one performed in the actual measurement but consisted of a larger amount of shorter trials (*n* trials = 24; see Section 5 (Method) for a detailed account). Due to technical difficulties, only a subset (*N* = 39) of our subjects participated in these assessments.

Task performance declined in both experiments with speech intelligibility, recognizable by a decrease in the mean hit rate. A one‐way repeated measures ANOVA across the three conditions revealed a main effect for Study#1 (*F*(2, 48) = 44.583, *p*_*ggeisser*_ = 7.35e^−09^, *η*_*p*_^*2*^ = 0.65) and Study#2 (*F*(2, 26) = 24.536, *p* = 1e^−06^, *η*_*p*_^*2*^ = 0.654). Comparing the different vocoding levels with each other showed higher hit rates for unvocoded stimuli than for stimuli vocoded with 7‐Channels (Study#1, *z*(24) = 2.916, *p*_*fdr*_ = .0035, *d* = 0.853; Study#2, *z*(13) = 2.566, *p*_*fdr*_ = .0102, *d* = 1.39) or 3‐Channels (Study#1, *z*(24) = 3.955, *p*_*fdr*_ = 7.7e^−05^, *d* = 2.151; Study#2, *z*(13) = 2.720, *p*_*fdr*_ = .0065, *d* = 2.280). Whereas stimuli vocoded with 7‐Channels showed higher hit rates than stimuli vocoded with 3‐Channels (Study#1, *z*(24) = 3.955, *p*_*fdr*_ = .0002, *d* = 1.491; Study#2, *z*(13) = 2.572, *p*_*fdr*_ = .0101, *d* = 1.265). Across all conditions hit rates differed significantly from chance (Study#1, Figure 1b; Study#2, Figure 1c): for unvocoded speech (Study#1, *z*(24) = 4.838, *p*_*fdr*_ = 3.932e^−06^; Study#2, *z*(13) = 3.742, *p*_*fdr*_ = .0005), for seven vocoding channels (Study#1, *z*(24) = 4.483, *p*_*fdr*_ = 1.103e^−05^; Study#2, *z*(13) = 3.355, *p*_*fdr*_ = .0011) and for three vocoding channels (Study#1, *z*(24) = 3.625, *p*_*fdr*_ = .0003; Study#2, *z*(13) = 3.105, *p*_*fdr*_ = .0019). This shows that while speech comprehension gradually decreases with increases in vocoding, speech was still intelligible even when only 3‐Channels were used to vocode the presented audio files.

### 2.2 Speech‐brain coherence declines with speech intelligibility

**FIGURE 2:**
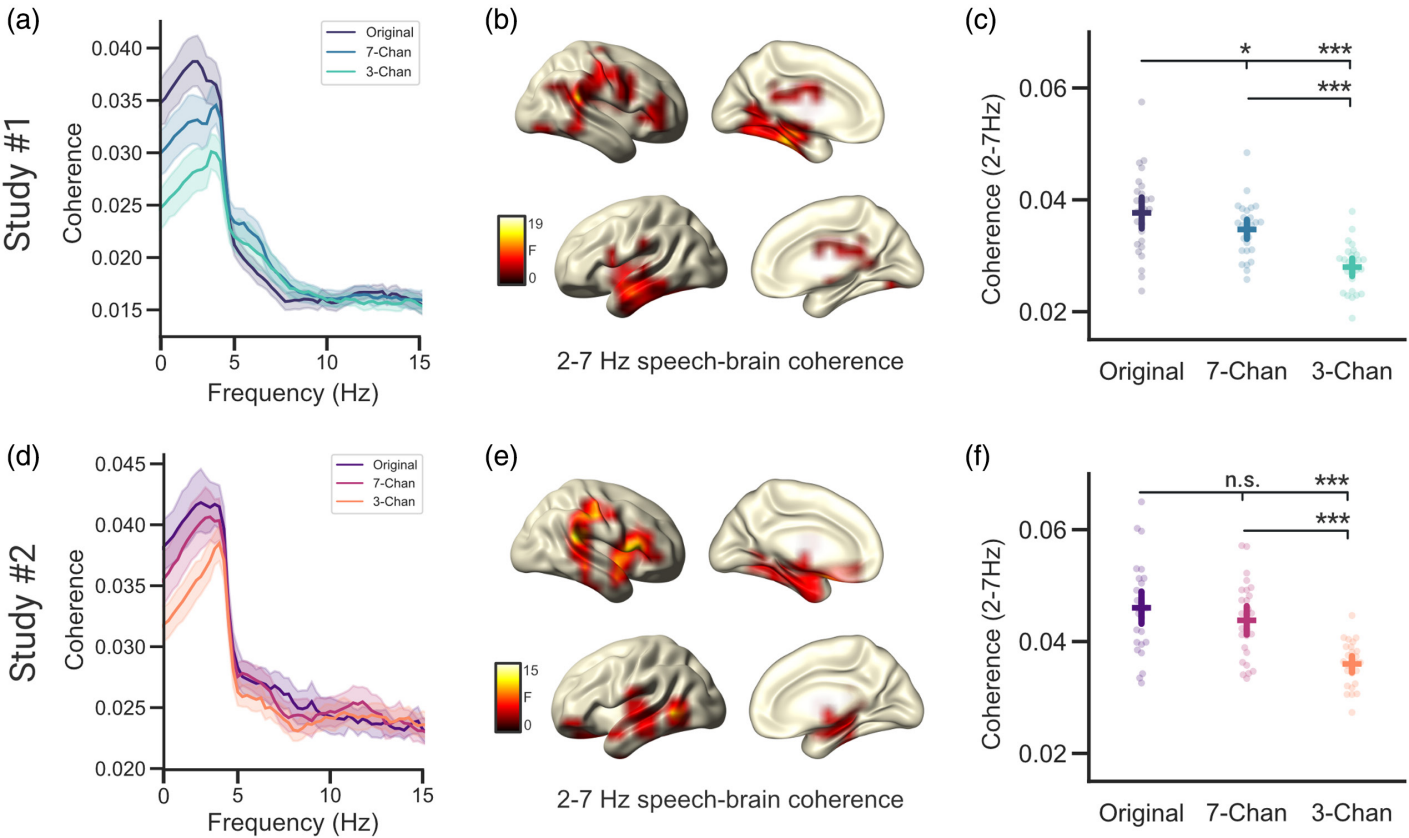
Speech‐brain coherence declines with speech intelligibility. (a,d) Speech‐brain coherence spectra for the three conditions averaged across all virtual channels. (b,e) Source localizations of degradation effects on speech‐brain coherence (2–7 Hz) during acoustic stimulation across three conditions (Original, 7‐Channels, and 3‐Channels) in bilateral temporal and medial frontal regions. (c,f) Individual coherence estimates (averaged) of the three vocoding conditions extracted at virtual channels showing a significant difference using a cluster‐corrected permutation test. Chan, Channels. Bars represent 95% confidence intervals, *p*_fdr_ < .05*, *p*_fdr_ < .001***.

To investigate how a loss of speech intelligibility via noise‐vocoding influences the neural dynamics of speech tracking we measured the coherence between the speech envelope and the related cortical activity (see coherence spectra in Figure 2a).

Comparisons of the coherence spectra across the three conditions (Original, 7‐Channels, and 3‐Channels) using a cluster‐corrected repeated measures ANOVA, revealed a significant difference in the low‐frequency range (averaged between 2 and 7 Hz) for both Study#1 (*p* = .0004) and Study#2 (*p* = 9e^−05^). This difference was strongest in right superior temporal gyrus for both Study#1 and #2. Both in Study#1 and #2 listening to the unaltered audio resulted in the strongest speech‐brain coherence, while the stimuli with the lowest intelligibility (3‐Channels; see Figure 1b,c) elicited the weakest coherence (Figure 2c,f). Listening to the unaltered (“Original”) audio files elicited stronger speech‐brain coherence than listening to speech vocoded with 7‐Channels in Study#1 (*t*(27) = 2.519, *p*_*fdr*_ = .018, *d* = 0.467) but not in Study#2 (*t*(26) = 1.425, *p*_*fdr*_ = .166, *d* = 0.307). However, listening to the unaltered (“Original”) audio files elicited a stronger coherence than listening to speech in the 3‐Channel condition (Study#1, *t*(27) = 6.083, *p*_*fdr*_ = 3e^−06^, *d* = 1.623; Study#2, *t*(26) = 7.451, *p*_*fdr*_ = 1.959e^−07^, *d* = 1.787). Listening to the 7‐Channels condition elicited higher levels of speech‐brain coherence than listening to the 3‐Channels condition (Study#1, *t*(27) = 6.238, *p*_*fdr*_ = 3e^−06^, *d* = 1.446; Study#2, *t*(26) = 7.021, *p*_*fdr*_ = 2.802e^−07^, *d* = 1.599).

In sum, these results show that both intermittent and continuous degradation similarly affect low‐frequency speech‐brain coherence. In both experimental designs, speech‐brain coherence decreased as speech became less intelligible. Comparing the decrease in coherence through vocoding across studies revealed that coherence decreased similarly across both studies (*U* = 297, *p* = .175, *r* = .214). At first glance, these results are in conflict with a previous analysis of Study#1 (Hauswald et al., 2022). The main difference between the previous and the current analysis of Study#1 can primarily be attributed to different filter settings (lower cut‐off for the high‐pass filter in the current analysis) during preprocessing that affected the offset and exponent of the speech‐brain coherence spectrum differently (see Section 3: Discussion). In the present study, these changes were applied to allow for better modeling of the periodic and the aperiodic components of the coherence spectrum. A specific separation of periodic and aperiodic components of the coherence spectrum is important as differences in aperiodic other than periodic components are likely generated by differences in signal‐to‐noise ratio between speech and brain activity (see Supplementary Material S1; for an analysis using simulated signals). Crucially, further analysis of these components showed that the aperiodic components explain most of the variance (Offset/Exponent; Study#1, *r* ^*2*^ = .83/.67; Study#2, *r* ^*2*^ = .36/.32) of the averaged (2–7 Hz) low‐frequency speech‐brain coherence in both studies (see Figure S3). This illustrates that analyzing coherence differences in a band‐limited range may be strongly influenced by aperiodic differences that do not necessarily reflect neural tracking of sound or linguistic information in the relevant frequency range. Depending on the filter settings, these aperiodic components may heavily impact the results. This observation is especially important for investigations that focus on slow and infraslow modulations and highlights the necessity to separate periodic from aperiodic contributions.

### 2.3 Declining speech intelligibility increases the center frequency of neural speech tracking along with a sharper tuning

**FIGURE 3:**
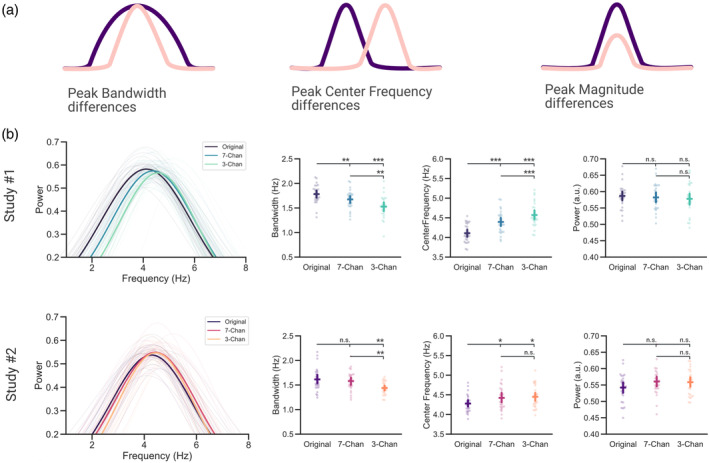
Declining speech intelligibility increases the center frequency of neural speech tracking along with a sharper tuning (a) Peak parameters influencing a significant coherence difference across experimental conditions. (b) The averaged relative magnitude, center frequencies, and bandwidth of peaks extracted from the coherence spectra for each subject were compared across three conditions (Original, 7‐Channels, and 3‐Channels). Chan, Channels. Bars represent 95% confidence intervals, *p*_fdr_ < .05*, *p*_fdr_ < .01**, *p*_fdr_ < .001***.

Both the speech envelope and electrophysiological signals (recorded using EEG/MEG) are characterized by an overall 1/f‐like spectrum (Pritchard, 1992; Voss & Clarke, 1975). This appears to also be evident in the coherence estimation between both signals (independent of the speech‐relevant peak at low frequencies; see Figure 2a,d). To quantify relevant aspects of the periodic components of speech tracking we extracted the most prominent peaks of the coherence spectra in the low‐frequency range across all virtual channels in which we observed a significant coherence difference across vocoding levels (see Figure 2b,e). This was operationalized by using *FOOOF* (Donoghue et al., 2020) to first flatten the coherence spectrum and then compute Gaussian model fits to extract peaks (see Figure S5 for a depiction of the parametrized grand average spectra). For each subject, the average relative magnitude of the coherence peak, the bandwidth (~tuning), and center frequency of the extracted peaks (Figure 3) were computed and compared within subjects and across the three conditions (Original, 7‐Channels, and 3‐Channels) using a repeated measure ANOVA.

This analysis showed that the actual magnitude of the extracted peaks did not differ across the three vocoding conditions in both studies (Study#1, *F*(2, 54) = 0.522, *p* = .596, *η*_*p*_^*2*^ = 0.019; Study#2, *F*(2, 50) = 2.18, *p* = .124, *η*_*p*_^*2*^ = 0.08). However, we noticed a significant difference across the center frequencies of the detected peaks over the three conditions in both studies (Study#1, *F*(2, 54) = 48.628, *p* = 8.365e^−13^, *η*_*p*_^*2*^ = 0.643; Study#2, *F*(2, 50) = 5.28, *p* = .008, *η*_*p*_^*2*^ = 0.175). Comparing the different vocoding levels with each other showed lower center frequencies for unvocoded stimuli than for stimuli vocoded with 7‐Channels (Study#1, *t*(27) = −7.122, *p*_*fdr*_ = 1.753e^−07^, *d* = −1.271; Study#2, *t*(25) = −2.756, *p*_*fdr*_ = .016, *d* = −0.613) and with 3‐Channels (Study#1, *t*(27) = −8.797, *p*_*fdr*_ = 6.18e^−09^, *d* = −1.918; Study#2, *t*(25) = −2.946, *p*_*fdr*_ = .0161, *d* = −0.7). The two vocoding conditions did differ significantly from each other in Study#1 (*t*(27) = −3.227, *p*_*fdr*_ = 3.273e^−03^, *d* = −0.544) but not in Study#2 (*t*(25) = −0.114, *p*_*fdr*_ = .91, *d* = −0.023) with lower center frequencies for speech vocoded with 7‐Channels compared to speech vocoded with 3‐Channels.

For the bandwidth of the detected peaks, differences across the three conditions were also observed both in Study#1 (*F*(2, 54) = 18.808, *p* = 6.329e^−07^, *η*_*p*_^*2*^ = 0.411) and Study#2 (*F*(2, 50) = 5.444, *p* = .007, *η*_*p*_^*2*^ = 0.179). In the continuous design, (Study#1) the tuning bandwidth for unvocoded stimuli was broader than for stimuli vocoded with 7‐Channels (*t*(27) = 3.219, *p*_*fdr*_ = .003, *d* = 0.666) and with 3‐Channels (*t*(27) = 5.196, *p*_*fdr*_ = 5.4e^−05^, *d* = 1.422). In the intermittent design (Study#2), the direction of the effect was similar, yet only significant for the difference between unvocoded speech and speech vocoded with 3‐Channels (*t*(25) = 3.398, *p*_*fdr*_ = .007, *d* = 0.983) and not for the difference between unvocoded speech and speech vocoded with 7‐Channels (*t*(25) = 0.699, *p*_*fdr*_ = .491, *d* = 0.201). Speech vocoded with 7‐Channels had a broader tuning bandwidth than speech vocoded with 3‐Channels across both studies (Study#1, *t*(27) = 3.592, *p*_*fdr*_ = .002, *d* = 0.758; Study#2, *t*(25) = 2.668, *p*_*fdr*_ = .02, *d* = 0.774).

**FIGURE 4:**
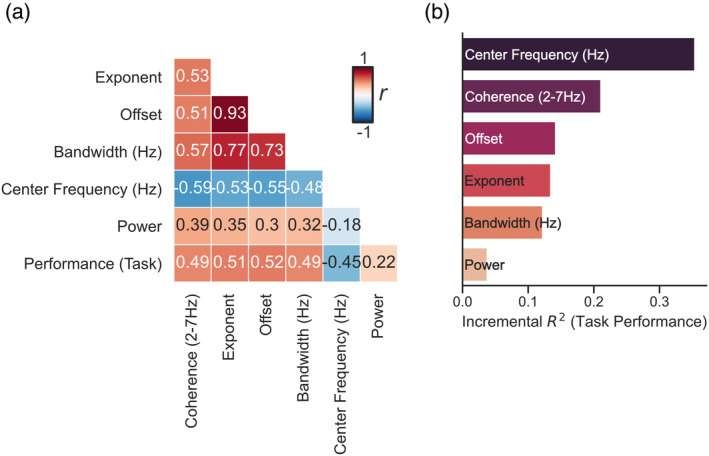
The center frequency of neural speech tracking explains the most unique variance in task performance (a) Correlation matrix of all features extracted from the speech‐brain coherence spectra obtained using repeated measures correlations across experimental conditions. (b) Dominance analysis reveals that center frequency explains the most unique variance in task performance of all parameters extracted from the speech‐brain coherence spectra.

In sum, these results show that intermittent and continuous degradation similarly affect the periodic components of speech‐brain coherence that are putatively reflective of neural speech tracking. Interestingly, the difference between speech tracking across different levels of intelligibility was not driven by the relative height of the peak in the coherence spectrum, but rather by a sharper tuning (Figure 4; bandwidth) combined with an increase in center frequencies of the coherence spectra (Figure 4; center frequency).

### 2.4 The center frequency of neural speech tracking explains the most unique variance in task performance

In order to better understand how the different parameters extracted from the coherence spectra are related to the task performance of our participants a repeated measures correlation for all extracted parameters was performed (Bakdash & Marusich, 2017). Behavioral data were only available for a subset of the participants we measured in the MEG (*N*_*Study#1*_ = 24; *N*_*Study#2*_ = 9). The analysis relating task performance to recorded brain activity was therefore only performed for a subset of subjects in Study#1. The results show that all parameters are to some degree significantly related (*p* < .05) and are all (apart from the relative magnitude of the coherence; *p* = .122) also significantly correlated with the observed task performance (see Figure 4a). Due to the partly very strong relationships between the parameters it is difficult to disentangle the independent contributions of a parameter on the observed task performance. To determine the relative importance of the predictors on the task performance we used dominance analysis (Azen & Budescu, 2003). Dominance analysis can be used to determine the incremental predictive validity of each predictor through directly comparing each predictor with all other predictors (Braun et al., 2019). We applied this analysis directly on the correlation matrix found in Figure 4a as suggested in (Laguerre, 2021) to determine the incremental *R* ^*2*^ of the parameters on the observed task performance. This analysis showed that center frequency explains the most unique variance in task performance of all parameters extracted from the speech‐brain coherence spectra (*R* ^*2*^ = .354).

### 2.5 Neural speech tracking shifts from syllabic to modulation rate as speech intelligibility decreases

**FIGURE 5:**
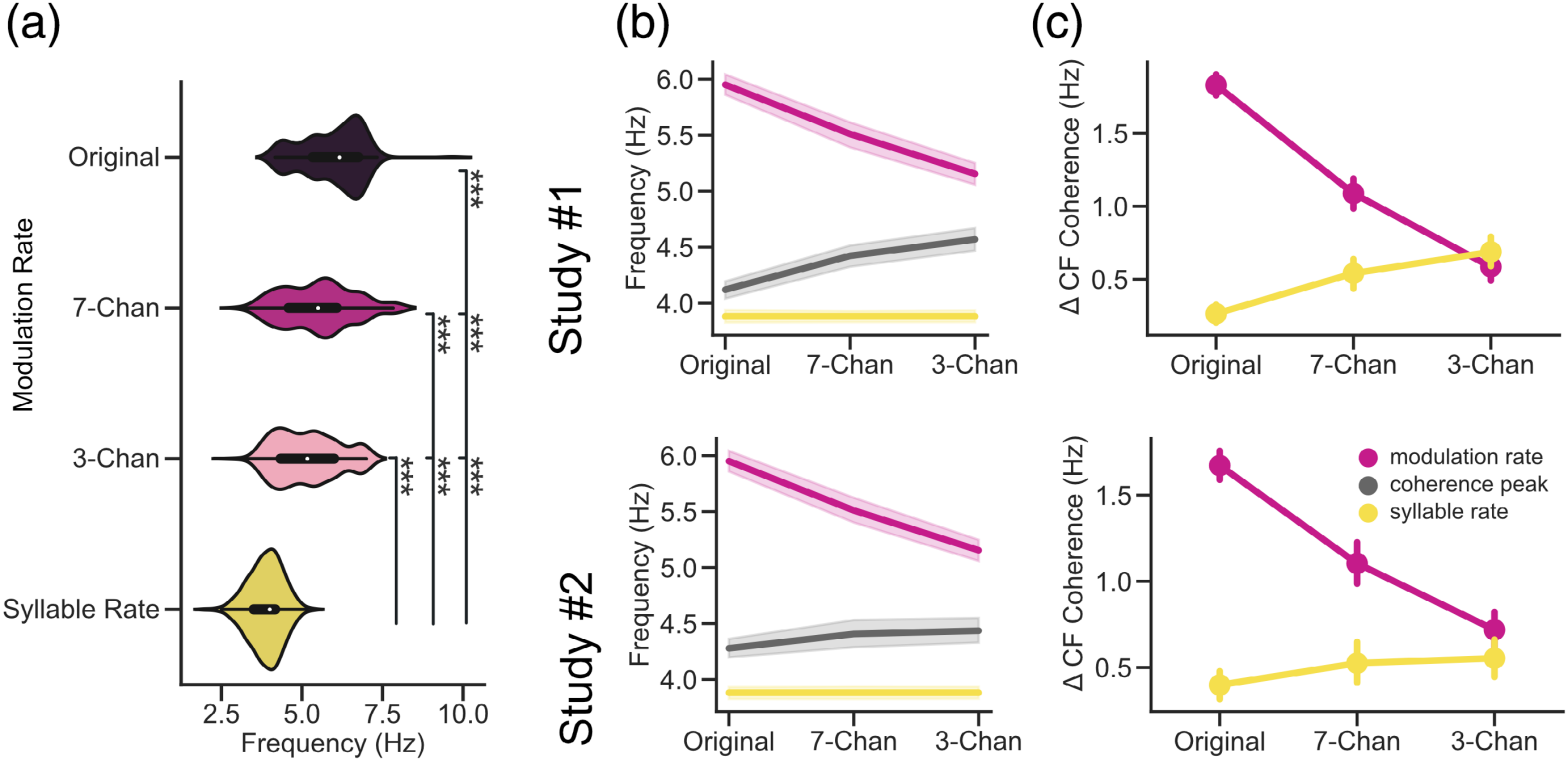
Neural speech tracking shifts from syllabic to modulation rate as speech intelligibility decreases (a) Center frequencies extracted from the acoustic envelopes of clear and vocoded speech (pink) and the syllabic rate (yellow). Although, we generally noted a strong overlap across the modulation spectra (see Figure 1a) the extracted modulation rates of the acoustic envelopes differed not only significantly from the syllabic rate, but also across vocoding levels. (b) The center frequency of speech‐brain coherence increases as intelligibility decreases, approaching the modulation rate of speech (pink; syllable rate displayed in yellow). (c) The absolute difference between center frequency speech‐brain and modulation and syllable rate shows a shift from syllabic to modulation rate as speech Intelligibility decreases. Chan, Channels. Bars represent 95% confidence intervals, *p*_fdr_ < .001***.

As speech intelligibility decreases we noted an increase in the center frequencies of speech‐brain coherence. Furthermore, we found that center frequency uniquely explains most of the variance related to task performance. We also extracted the center frequencies of the modulation spectra from the acoustic envelopes of the audiobook for the three conditions (Original, 7‐Channels, and 3‐Channels using the same method as in (Ding et al., 2014); see Figure 5a) and computed the realized syllable rate of the presented audiobook (de Jong & Wempe, 2009). Although, there was generally a strong overlap over the modulation spectra of the speaker across vocoding levels (see Figure 1a), a one‐way repeated measures ANOVA across the extracted center frequencies and the syllable rate of the audio signal revealed a significant main effect (*F*(3, 1098) = 454.104, *p* = 2.68e^−175^, *η*_*p*_^*2*^ = 0.554). The center frequencies and the syllabic rate across all conditions differed significantly (see Figure 5 & Tables S1 and S2 for a related post‐hoc analysis). The rate at which the syllables were produced (*Median* = 4 Hz) was lower than the center frequencies of the modulation spectra of the audio signal 3‐Channels (*Median* = 5.16 Hz), 7‐Channels (*Median* = 5.5 Hz), and clear speech condition (*Median* = 6.16 Hz). The increase in center frequencies of speech‐brain coherence along with the differences in modulation and syllable rates suggests that the brain may be driven more by the acoustic or linguistic information encoded in the speech envelope depending on the signal quality. This is intuitive, as with increased vocoding it also becomes more difficult to extract linguistically meaningful information such as phrase boundaries, words, or syllables. This mainly leaves the modulation intensities of the acoustic speech envelope as an information source to the listener (that could be generated by syllables, phones, words, or other sounds unrelated to any linguistic information). The following analysis (in combination with Figure S4) aims at addressing this point more directly.

We calculated the absolute difference between the average modulation/syllable rate per vocoding condition and the individual center frequencies of speech‐brain coherence (see Figure 5b,c). We then compared the “absolute difference” between both conditions using a two‐way repeated measures ANOVA. This analysis revealed that there was a significant main effect for the factors tracking (modulation/syllable rate) in both studies (Study#1 *F*(1, 27) = 79.406, *p*_*ggeisser*_ = 1.58e^−9^, *η*_*p*_^*2*^ = 0.66; Study#2 *F*(1, 26) = 62.357, *p*_*ggeisser*_ = 2.26e^−8^, *η*_*p*_^*2*^ = 0.58). The absolute difference between neural speech tracking and the syllable rate (linguistic component) was overall lower compared to the modulation rate (acoustic component). There was also a significant main effect of Vocoding (Original, 7‐Channels, and 3‐Channels; Study#1 *F*(2, 54) = 2872.121, *p* = 5.85e^−29^, *η*_*p*_^*2*^ = 0.32; Study#2 *F*(2, 52) = 150,642.274, *p* = 3.86e^−58^, *η*_*p*_^*2*^ = 0.25). The absolute difference between neural speech tracking and the modulation/syllable rate decreased across vocoding levels. However, there was a significant interaction effect for the factors tracking (modulation/syllable rate) and vocoding (Original, 7‐Channels, and 3‐Channels) across both Studies (Study#1 *F*(2, 54) = 164.529, *p*_*ggeisser*_ = 3.66e^−23^, *η*_*p*_^*2*^ = 0.671; Study#2 *F*(2, 52) = 39.536, *p*_*ggeisser*_ = 6.62e^−11^, *η*_*p*_^*2*^ = 0.39). This suggests that while speech intelligibility decreases and less linguistically meaningful information is present, neural speech tracking starts to drift away from the syllabic rate toward the modulation rate of speech.

### 2.6 Modeling of subcortical activity reveals a predominant tracking of the modulation rate of speech

Recent studies using noninvasive electrophysiology have shown that auditory activity at putative subcortical processing stages can be measured for continuous complex natural sounds (such as speech; (Etard et al., 2019; Forte et al., 2017; Maddox & Lee, 2018; Polonenko & Maddox, 2021)). Furthermore, some studies suggest that this subcortical auditory activity can even be modulated by attention (Etard et al., 2019; Forte et al., 2017; Gehmacher et al., 2022). Interestingly, top‐down attentional modulations of auditory activity can already be detected at the hair cells in the inner ear measured as otoacoustic activity (faint sounds emitted by the outer hair cells; see (Köhler et al., 2021)). Other studies have shown that subcortical nuclei on the auditory pathway carry a behaviorally relevant role for speech recognition (medial geniculate bodies; (von Kriegstein et al., 2008)). Using a recently developed modeling procedure (Schmidt et al., 2020), we further aimed to investigate whether speech intelligibility already influences auditory processing at subcortical areas along the auditory pathway.

**FIGURE 6:**
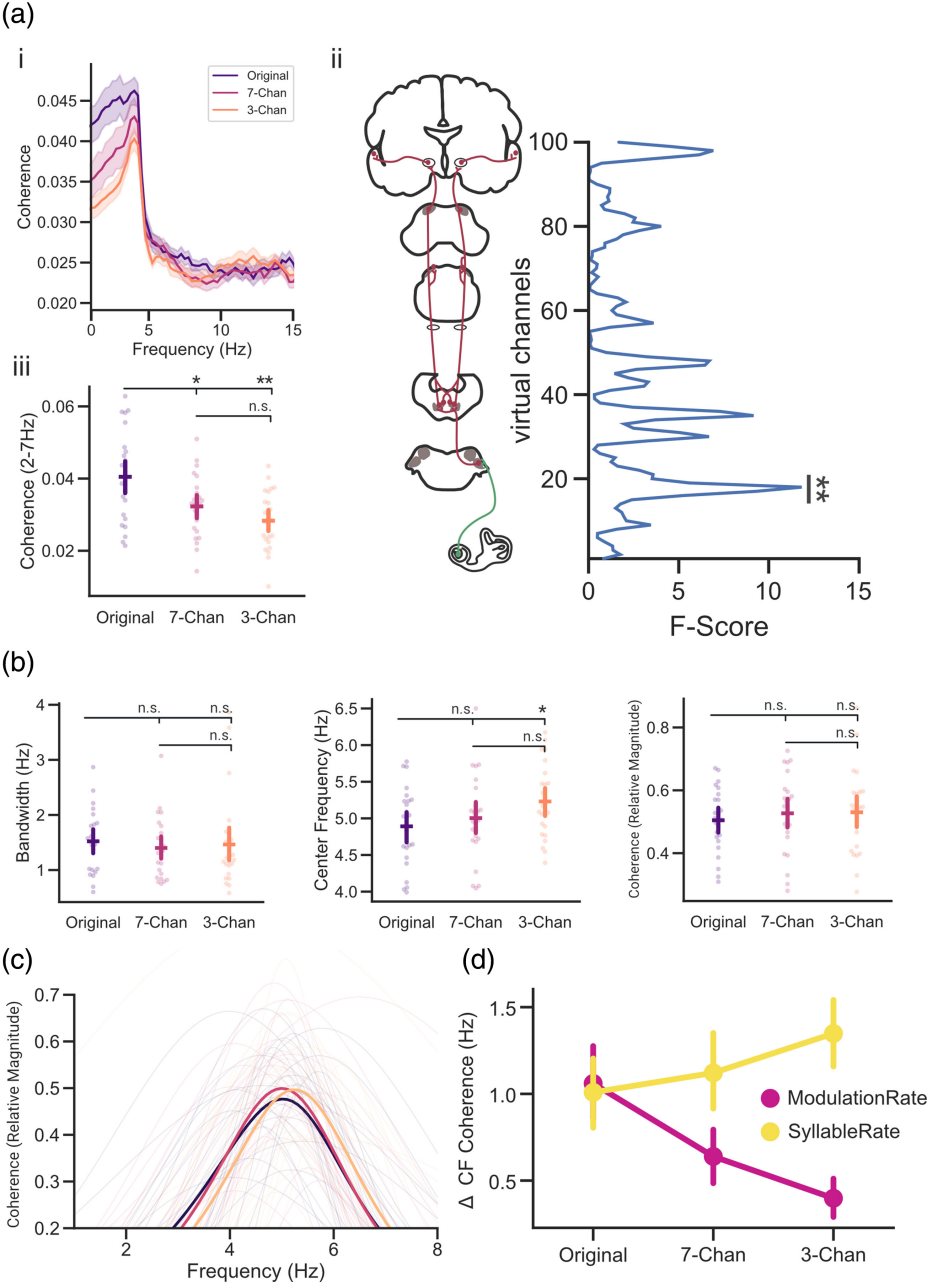
Modeling of subcortical activity reveals a predominant tracking of the modulation spectrum of speech (ai) Speech‐brain coherence spectra for the three conditions averaged across all virtual channels. (aii) The three conditions (Original, 7‐Channels, and 3‐Channels) differed significantly at virtual channels reflecting activity putatively related to the auditory nerve/cochlear nucleus (channels 16–20). (aiii) Individual coherence (2–7 Hz) extracted at channels 16–20 was highest for clear speech. (b,c) Peak height, center frequencies and bandwidth of peaks extracted from virtual channels (16–20) (d) Absolute Differences between the center frequencies of the coherence spectra and the syllable/modulation rate of speech. Chan, Channels. Bars represent 95% confidence intervals, *p*_fdr_ < .05*, *p*_fdr_ < .01**.

We used a localizer measurement (Schmidt et al., 2020) to compute individualized weights (per subject; note that the localizer was only available for Study#2). These weights reflect activity along the auditory hierarchy, resulting in 100 virtual channels ranging from the auditory nerve (channels 0–20) to early thalamo(−cortical) processing stages (channels 90–100). We then applied these weights (see Section 5.10: Method: Modeling of subcortical auditory activity) to the epoched data from Study#2 to infer activity along the auditory hierarchy (see spectral distribution in Figure 6a). A cluster‐corrected repeated measures ANOVA across the three conditions (Original, 7‐Channels, and 3‐Channels) and within subjects revealed a significant difference in the low‐frequency range (2–7 Hz) between virtual channels that are reflective of subcortical activity at early stages of auditory processing (putatively auditory nerve/cochlear nucleus, *p* = .0045). Listening to the unaltered (“Original”) audio files elicited higher speech‐brain coherence than listening to the 7‐Channels (*t*(24) = 3.2, *p*_*fdr*_ = .005, *d* = 0.798) and the 3‐Channels condition (*t*(24) = 4.282, *p*_*fdr*_ = .0008, *d* = 1.212). However, the two vocoding conditions did not differ significantly from each other (*t*(24) = 1.547, *p*_*fdr*_ = .135, *d* = 0.488).

We further investigated the periodic components that are reflective of speech tracking by extracting peaks from the coherence spectra to analyze the corresponding magnitude of the coherence peak, the bandwidth, and center frequencies. A repeated measures ANOVA across conditions (Original, 7‐Channels, and 3‐Channels) and within subjects revealed no significant differences for the relative magnitude of the coherence peak (*F*(2, 48) = 0.335, *p* = .717, *η*_*p*_^*2*^ = 0.014) and the bandwidth of the extracted peaks (*F*(2, 48) = 0.192, *p* = .826, *η*_*p*_^*2*^ = 0.008). However, significant differences were found across conditions for the center frequencies of the peaks (*F*(2, 48) = 3.213, *p* = .049, *η*_*p*_^*2*^ = 0.118). Listening to the unaltered (“Original”) audio files was associated with significantly lower center frequencies than listening to the 3‐Channel condition (*t*(24) = −3.062, *p*_*fdr*_ = .016, *d* = −0.664) but not than listening to the 7‐Channel condition (*t*(24) = −0.767, *p*_*fdr*_ = .450, *d* = −0.204) at subcortical processing stages. The two vocoding conditions did not differ significantly from each other (*t*(24) = −1.528, *p*_*fdr*_ = .21, *d* = −0.425).

We further calculated the absolute difference between the average modulation/syllable rate per vocoding condition and the individual center frequencies of speech‐brain coherence (see Figure 5) to detect whether the tracking of speech at early auditory processing stages could be closer related to the modulation rate of speech or the syllabic rate. We found that modeling of the related subcortical activity reveals predominantly a tracking of the acoustic modulation rate of speech (*F*(1, 24) = 16.14, *p* = .0005, *η*_*p*_^*2*^ = 0.19), contrary to the previous analysis mainly reflecting cortical effects (see Figure 5). However, similar to the previous analysis reflecting mainly cortical activity there was an interaction effect between tracking and vocoding, with decreasing intelligibility the center frequency of neural speech tracking approaches the modulation away from the syllabic rate (*F*(2, 48) = 10.696, *p* = .0004, *η*_*p*_^*2*^ = 0.16).

In sum, these results suggest that differences in speech tracking between clear and vocoded stimuli already arise at subcortical processing stages. This difference in neural speech tracking occurs at virtual channels that can be putatively associated with subcortical activity in the auditory nerve and cochlear nucleus. The extracted peaks at this level of processing did not differ significantly from each other regarding the relative peak height of the coherence (similar to cortical observations; see Figure 5) and their tuning width (different to cortical observations; see Figure 5). Yet, the center frequency shift of these peaks showed a similar effect when compared to cortical processing stages. As intelligibility decreased the absolute difference between the center frequencies and the modulation rate decreased, while the difference to the syllable rate increased steadily. However, contrary to the cortical recordings, tracking across different levels of intelligibility at subcortical processing stages was overall predominantly related to the modulation rate of speech instead of the syllabic rate (different to cortical observations; see Figure 5). This shows that although tracking at a subcortical level is dominated by lower‐level acoustic envelope modulations, intelligibility also influences these hierarchically early responses.

## 3 DISCUSSION

Speech tracking is modulated by the intelligibility of the sensory input. However, the pattern of that modulation—frequently operationalized by band‐limited coherence effects—is not consistent across studies (see e.g., (Ding et al., 2014; Hauswald et al., 2022; Luo & Poeppel, 2007)). This complicates a mechanistic understanding of how speech tracking actually supports speech comprehension. Applying a method to separate periodic from aperiodic components in the coherence spectrum, our results yield a differentiated picture, indicating that intelligibility affects tuning‐width and center frequency of the periodic components in the low‐frequency range.

### 3.1 Band‐limited speech‐brain coherence declines with speech intelligibility

Here, we investigated the effects degraded speech has on the neural dynamics of speech tracking using data from two slightly different experimental paradigms. In Study#1 speech was displayed continuously at one of three different levels of intelligibility (Original, 7‐Channels, and 3‐Channels; ~15 s–3 min). In Study#2 segments of degraded speech (7‐Channels and 3‐Channels; ~6–18 s) were embedded in a clear audio stream (Original; ~1–3 min) as both studies produced comparable results, they will be discussed together. We observed in accordance with previous results (Doelling et al., 2014; Gross et al., 2013; Keitel et al., 2018; Peelle et al., 2013) that low‐frequency speech‐brain coherence declines with a decrease in intelligibility. However, other studies have reported a variety of partly contradicting results (Ding et al., 2014; Hauswald et al., 2022; Song & Iverson, 2018). Our present results show that the reported band‐limited coherence spectra are very strongly related to the underlying aperiodic components in the spectrum (see Supplementary Material). Since the field is mostly interested in neural tracking of (relatively) periodic speech features around the syllable rate, it is questionable whether band‐limited coherences without consideration of the aperiodic components are a viable measure for neural speech tracking.

### 3.2 Neural speech tracking shifts from syllabic to modulation rate as speech intelligibility decreases

Interestingly, in the investigation of spectral power differences in electrophysiological signals, a variety of contradicting results is also commonly reported for band‐limited effects. The present study suggests that this may be caused by the conflation of periodic (center frequency, power, bandwidth) and aperiodic (offset, exponent) properties of the underlying signal (Donoghue et al., 2020). This is deemed problematic as periodic and aperiodic components of the signal can be linked to a variety of different effects (Donoghue et al., 2020). Both the acoustic envelope of speech and electrophysiological measurements of neural activity possess an overall (aperiodic) 1/f‐like spectrum (Pritchard, 1992; Voss & Clarke, 1975). This 1/f‐like pattern is at times also found in the low‐frequency coherence/correlation spectrum between both signals (e.g. see (Ding et al., 2014; Gross et al., 2013; Hauswald et al., 2022)). We therefore decomposed the speech‐brain coherence spectra in their periodic and aperiodic components using *FOOOF* (Donoghue et al., 2020), to better understand the relationship between the intelligibility of speech and the related neural dynamics of speech tracking. Interestingly, these investigations revealed that the aperiodic components (offset and exponent) explained most of the variance observed in the coherence difference (at 2–7 Hz; see Figure 2) across vocoding levels (see Figure S2). This highlights the importance of separating periodic from aperiodic components in the speech‐brain coherence spectra, as we were primarily interested in investigating peaks in the coherence spectra (periodic components) that can be related to neural speech tracking. Further investigations of the periodic components of the low‐frequency coherence peak (center frequency, relative magnitude, bandwidth) revealed that there was no difference across vocoding levels in the relative magnitude of the coherence peak. Instead, the periodic differences in neural speech tracking were rather caused by a sharpening in the frequency tuning of the coherence peak of vocoded speech along with an increase in the center frequencies of the observed peaks (see Figure 3). We were able to link the increase in the center frequencies to a shift in tracking from higher‐level linguistic to lower‐level acoustic information of the speech stream.

Our analysis showed that neural tracking shifts from the syllabic (linguistic) to the modulation (acoustic) rate as intelligibility decreases. This shift between rates also intuitively makes sense, as with decreased intelligibility it also becomes more difficult to extract linguistically meaningful information such as phrase boundaries or syllables. This mainly leaves the modulation intensities of the acoustic speech envelope as an information source to the listener. As the acoustic modulation of speech is closely related to the production of syllables (Poeppel & Assaneo, 2020), investigations of neural speech tracking are typically not making a distinction between lower‐level acoustic and higher‐level linguistic information on the level of syllable processing. However, while the modulation rate (acoustic property) of speech appears to be exceptionally stable across languages and speaking conditions (Ding et al., 2017; Poeppel & Assaneo, 2020), the syllable rate (linguistic property) of speech differs depending on the language and the speaking conditions (Coupé et al., 2019; Jacewicz et al., 2009). This suggests that modulation rate and syllable rate are not terms that can be necessarily used interchangeably. Therefore, distinguishing these properties more clearly may also be important to gain a better understanding of the neural processes separating auditory processing disorders (e.g., hearing loss) from language processing disorders (e.g., developmental dyslexia), which has been difficult based solely on neural speech tracking. This difficulty may be linked to the variety of (partly contradicting) results within and across auditory/linguistic processing disorders that relate to the neural dynamics of speech tracking. While a recent study was able to link hearing loss to a relative increase in speech envelope tracking (compared to (age matched) normal hearing listeners (Decruy et al., 2020)), previous studies could not report enhanced envelope tracking in individuals with a hearing impairment (Mirkovic et al., 2019; Presacco et al., 2019). Related to language proficiency, similar inconsistencies are reported as non‐native speakers appear to show an increased envelope tracking compared to native speakers (Reetzke et al., 2021; Song & Iverson, 2018). On the other hand, individuals suffering from developmental dyslexia are reported to have lower synchronization with the speech envelope compared with neurotypical individuals (Molinaro et al., 2016). Using the approach proposed here of decomposing coherence spectra in their periodic and aperiodic components, it should be possible to gain a more fine‐grained view on the specific characteristics underlying the neural dynamics of speech tracking. This may help in the future to better differentiate the neural signatures of individuals suffering from auditory processing or language processing disorders.

### 3.3 Declining speech intelligibility goes along with a sharper frequency tuning

Apart from the intelligibility‐dependent changes in the center frequencies of the coherence peaks, we also noted a wider frequency tuning of speech tracking in clear as opposed to vocoded speech. The width of this frequency tuning decreased with a loss in intelligibility. As the syllabic rate of our speaker (~4 Hz) differed from the modulation rate of her speech stream (~5–6 Hz; see Figure 4), the narrowing in tuning may also be related to a loss in linguistically meaningful information. This might suggest that in situations where speech is clear, both linguistic (syllable rate) and acoustic information (modulation rate) were tracked resulting in an increased bandwidth covering all relevant frequencies. As speech becomes less intelligible and it becomes harder to extract linguistically meaningful information, the bandwidth of the coherence peak narrows around the higher frequencies of the residual acoustic modulation of speech. Furthermore, previous studies have shown that auditory selective attention effects may arise from an enhanced tuning of receptive fields of task‐relevant neural populations (Ahveninen et al., 2006; Atiani et al., 2009). Therefore, the observed narrower frequency tuning could also be related to enhanced top‐down auditory attention processes (Atiani et al., 2014) in situations where listening becomes more challenging.

### 3.4 Influences of aperiodic components on the neural dynamics of speech tracking

Investigating the parameters related to low‐frequency peaks in measurements of speech‐brain coherence is offering a new and unique perspective to better understand the neural dynamics underlying speech tracking. However, we also noticed that band‐limited differences in the speech‐brain coherence spectra are strongly related to the underlying aperiodic components. This highlights the importance to separate periodic from aperiodic components, as periodic and aperiodic components can be linked to a variety of different effects (Donoghue et al., 2020). Commonly, aperiodic components of most signals have been considered as noise and as such are often just removed from the overall signal. Especially for low‐frequency activity this can be easily achieved by spectrally normalizing (whitening) the signal via filtering (e.g., see (Demanuele et al., 2007)). Different choices in filter settings, however, can also generally accentuate different properties of a signal. For instance, in this study we reanalyzed data from a recent study (Study#1; (Hauswald et al., 2022)) using a larger time window for the coherence estimation (4 s instead of 2 s; to obtain a better frequency resolution for low‐frequency speech tracking) and a lower cut‐off for the high‐pass filter (0.1 Hz instead of 1 Hz). These changes were intended to improve the model fit of *FOOOF* for the low‐frequency coherence spectra, but also resulted in a different pattern for low‐frequency speech‐brain coherence (compare Figure 2a‐c with figure 2a,b in Hauswald et al., 2022). The previous analysis of Study#1 (Hauswald et al., 2022) showed that neural speech tracking increases for mild decreases in intelligibility (putatively driven by an increased listening effort) and then decreases as speech becomes increasingly unintelligible. We now show that low‐frequency speech tracking gradually decreases with intelligibility. This difference was mainly driven by changes in filter settings accentuating different properties of the signal by putatively differently influencing the 1/f‐like pattern of low‐frequency speech‐brain coherence. Similar to the analysis of power spectral densities, 1/f‐like patterns in the coherence spectra also appear to play a striking role when computing statistics across experimental conditions (see S2 for a comparison of slope and offset for the data analyzed in the present study). Furthermore, aperiodic parameters were correlated with behavioral performance (see Figure 4; across a subset of subjects) and explained most of the variance of the speech‐brain coherence spectrum (see Figure S3; across all subjects). However, the extent to which aperiodic activity explained variance in the speech‐brain coherence spectrum varied depending on running the analysis on the whole sample (see Figure S3) or on a subset (see Figure 4). Therefore, additional studies using a larger sample might be needed to better understand the role of aperiodic components of the speech‐brain coherence spectrum. However, whether or not 1/f‐like patterns carry (in general) meaningful information is heavily debated. Nevertheless, recent studies have shown that 1/f‐like patterns in electrophysiological power spectra can change both dependent on trait‐like factors (age (Voytek et al., 2015), ADHD (Robertson et al., 2019), and schizophrenia (Molina et al., 2020)) and state‐like factors (e.g. differences over cognitive and perceptual states (He et al., 2010; Podvalny et al., 2015)). This suggests a physiologically meaningful underpinning of 1/f‐like neural activity. However, interpretations related to the aperiodic patterns found in low‐frequency speech‐brain coherence go beyond the scope of the present study, as we were mainly focused on the distinction between the processing of the syllabic rate and the modulation rate of speech related to peaks in the speech‐brain coherence spectra (periodic components). Perhaps aperiodic components of speech‐brain coherence could be modulated by slower components in the speech stream reflecting higher‐level information (e.g., sentence or phrasal information), that become increasingly lost with less intelligibility. Addressing this question should be the topic of future investigations using paradigms in which these features are parametrically controlled (Ding et al., 2016). However, the present study illustrates that analyzing coherence in a band‐limited range, even though more or less explicitly assumed, may not reflect neural tracking of sound or linguistic information in the relevant frequency range. Instead, depending on the filter settings, the aperiodic components may heavily impact the results. This is especially important for investigations that focus on slow and infraslow modulations.

### 3.5 Modeling of subcortical activity reveals a predominant tracking of the modulation spectrum of speech

Previous research has shown that not only cortical, but also subcortical regions play an important role in language processing (Diaz et al., 2012). These subcortical regions appear to be even behaviorally relevant for speech recognition (medial geniculate bodies; (von Kriegstein et al., 2008)). Here, we generated individualized spatial filters reflective of subcortical auditory processing using a localizer measurement (Schmidt et al., 2020). In principle, these filters can be applied to a separate measurement to infer subcortical auditory activity. Using this modeling procedure, we aimed to investigate whether differences in speech intelligibility can already be observed at putative subcortical processing stages. Similarly to the activity from cortical processing stages, we noticed a shift of the center frequency of the extracted peaks. As intelligibility decreased, the center frequencies of the detected peaks increased steadily. However, contrary to the cortical recordings, our results showed that the center frequencies of the speech‐brain coherence peaks (reflecting neural speech tracking) across different levels of intelligibility at subcortical processing stages were predominantly related to the modulation rate of speech opposed to the syllabic rate. This shows that although tracking at a subcortical level is overall higher for the low‐level acoustic envelope modulation, intelligibility also influences these hierarchically early responses (see Figure 5d). This highlights the potentially important yet often overlooked role of subcortical nuclei in speech and language processing.

## 4 CONCLUSION

In this study, we introduce a novel way to investigate neural speech tracking by utilizing an approach recently introduced to parametrize electrophysiological power spectra (Donoghue et al., 2020). Our results show that cortical regions mostly track the syllable rate, whereas subcortical regions are driven by the acoustic modulation rate. Furthermore, the less intelligible speech becomes, the more dominant the tracking of the modulation rate becomes. Our study underlines the importance of making a distinction between the acoustic modulation and syllable rate of speech and provides novel possibilities to better understand differences between auditory processing and speech/language processing disorders. In general, parametrization of coherence spectra may offer a new and unique perspective to investigate the parameters that drive neural speech tracking across a variety of listening situations.

## 5 METHOD

### 5.1 Subjects

Twenty‐eight individuals participated in Study#1 (female = 17, male = 11). Mean age was 23.82 years (standard deviation, *SD* = 3.71) with a range between 19 and 37 years. In Study#2 27 individuals participated (female = 11, male = 16). Due to technical difficulties one subject was removed from Study#2. Mean age was 23.38 years (*SD* = 4.15) with a range between 19 and 38 years. Across both studies we recruited only German native speakers and people who were suitable for MEG recordings, that is, without nonremovable ferromagnetic metals in or close to the body. Participants provided informed consent and were compensated monetarily or via course credit. Participation was voluntary and in line with the declaration of Helsinki and the statutes of the University of Salzburg. The study was approved by the ethical committee of the University of Salzburg.

### 5.2 Stimuli

For the MEG recording, audio files were extracted from audio–visual recordings of a female speaker reading Goethe's “Das Märchen” (“The Tale”; 1795). In Study#1, lengths of 12 stimuli varied between approximately 15 s and 3 min, with two stimuli of 15, 30, 60, 90, 120, and 150 s, and six of 180 s. Stimuli were presented in three blocks with four stimuli in each block. In Study#2, two or three segments of degraded speech (7‐Channels and 3‐Channels; 4.8–21.6 s) were embedded in 15 clear audio streams. The lengths of the 15 stimuli varied between 60 s and 3 min with two stimuli of 60, 90, 120, and 9 of 180 s. Stimuli were presented in five blocks with three stimuli in each block. In both studies, each stimulus ended with a two‐syllable noun within the last four words. In order to keep participants' attention on the stimulation, we asked participants after each stimulus to choose from two presented two‐syllable nouns, the one that had occurred within the last four words of a sentence. The sequence of all the audio stimuli was randomized across participants, not following the original storyline of the audiobook. The syllable rate of the stimuli varied between 3.1 and 4.3 Hz with a median of 4 Hz (estimated using Praat (de Jong & Wempe, 2009)).

### 5.3 Vocoding

Noise‐vocoding of all audio stimuli was done using the vocoder toolbox for MATLAB (Gaudrain, 2016), and we created conditions with 7‐ and 3‐Channels (Figure 1a). Vocoding for both studies was performed as described in (Hauswald et al., 2022). For the vocoding, the waveform of each audio stimulus was passed through two Butterworth analysis filters (for 7‐ and 3‐Channels) with a range of 200–7000 Hz representing equal distances along the basilar membrane. Amplitude envelope extraction was done with half‐wave rectification and low‐pass filtering at 250 Hz. The envelopes were then normalized in each channel and multiplied with the carrier. Then, they were filtered in the band and the RMS of the resulting signal was adjusted to that of the original signal filtered in that same band. Auditory stimuli were presented binaurally using MEG‐compatible pneumatic in‐ear headphones (SOUNDPixx, VPixx technologies).

### 5.4 Behavioral assessment

Due to the low number of behavioral responses from the MEG part, we added an additional behavioral assessment. For Study#1 and Study#2, 24 audio files were created from recordings of another female native German speaker reading Antoiné St. Exupery's “The little prince” (1943). Each stimulus contained one sentence (length between 2 and 15 s) and was either presented unvocoded with 7‐Channel vocoding or 3‐Channel vocoding. For Study #1, the stimuli in the vocoding condition were vocoded from start to the end; for Study #2, the stimuli were vocoded only in the last 0.6–5 s. Comparable to both MEG experiments, the stimuli also ended with a two‐syllable noun within the last four words, and participants were asked to choose the last noun they heard between two nouns on the screen. The sequence of all audio stimuli was random across the participants, not following the storyline. In each study, the hit rates across the three vocoding conditions were compared using one‐way repeated measures ANOVAs. Post‐hoc analysis was performed using FDR (Benjamini & Hochberg, 1995) corrected Wilcoxon signed‐rank tests (as the assumptions for paired samples *t*‐tests were violated).

### 5.5 Data acquisition

Data acquisition and parts of the data analysis for Study#1 and #2 closely resemble, with minor exceptions, the one described in two previous studies (Hauswald et al., 2018, 2020). Magnetic brain activity was recorded using a 306‐channel whole head MEG system (TRIUX, Elekta Oy) with a sampling rate of 1 kHz for the main experiments (Study#1 and Study#2) and with a sampling rate of 10 kHz for the brainstem localizer in Study#2 (see *Backward Modeling* for further information). The system consists of 204 planar gradiometers and 102 magnetometers. Before entering the magnetically shielded room (AK3B, Vakuumschmelze), the head shape of each participant was acquired with >300 digitized points on the scalp, including fiducials (nasion, left and right preauricular points) with a Polhemus FASTRAK system (Polhemus). The auditory brainstem response was measured with a single electrode located on FpZ based on the electrode placement of the international 10–20‐System (Klem et al., 1999). A ground electrode was placed on the forehead at midline and a reference on the clavicle bone of the participants.

### 5.6 Data analysis

#### 5.6.1 Preprocessing

All data analysis steps for Study #1 and #2 were performed similarly and are therefore reported together. The acquired data were Maxwell filtered using a Signal Space Separation (SSS) algorithm (Taulu & Simola, 2006) implemented in the Maxfilter program (version 2.2.15) provided by the MEG manufacturer to remove external magnetic interference from the MEG signal and realign data to a common standard head position (−trans default Maxfilter parameter). The Maxwell‐filtered and continuous data were then further analyzed using the FieldTrip toolbox (Oostenveld et al., 2011) and custom‐built Matlab routines. First, the data were high‐pass filtered at 0.1 Hz using a finite impulse response (FIR) filter (Kaiser window). For extracting physiological artifacts from the data, 50 independent components were calculated from the filtered data, using the *runica* method implemented in the FieldtripTrip toolbox (Oostenveld et al., 2011). Via visual inspection, the components showing eye movements and heartbeats were removed from the data. On average across studies, three components were removed per subject (*SD* = 1). Then, trials related to each of the three conditions (Original, 7‐Channels, and 3‐Channels) were defined. The acoustic speech envelope was extracted and aligned with the measured MEG data (Hauswald et al., 2022). Afterwards data were cut into segments of 4 s to increase signal‐to‐noise ratio.

### 5.7 Source analysis

Anatomical template images were warped to the individual head shape and brought into a common space by co‐registering them based on the three anatomical landmarks (nasion, left and right preauricular points) with a standard brain from the Montreal Neurological Institute (MNI, Montreal, Canada) (Mattout et al., 2007). Afterwards a single‐shell head model (Nolte, 2003) was computed for each participant. As a source model, a grid with 1 cm resolution and 2982 virtual channels based on an MNI template brain was morphed into the brain volume of each participant. This allows group‐level averaging and statistical analysis as all the grid points in the warped grid belong to the same brain region across subjects. Common linearly constrained minimum variance (LCMV) beamformer spatial filters (Van Veen et al., 1997) were then computed on the preprocessed MEG data and applied to project the single‐trial time series into source space. The number of epochs across conditions was equalized (by the lowest number of epochs across conditions within each study). We applied a frequency analysis to the 4‐s segments of all three conditions (Original, ‐Channels, and 3‐Channels) calculating multi‐taper frequency transformation (dpss taper: 0–25 Hz in 0.25 Hz steps, 4 Hz smoothing, no baseline correction). For the coherence calculation between each virtual sensor and the acoustic speech envelope, 0.25‐Hz frequency steps were chosen. Then, the coherence between activity at each virtual sensor and the acoustic speech envelope during acoustic stimulation in the frequency spectrum was calculated and averaged across trials. We refer to the coherence between acoustic speech envelope and brain activity as neural speech tracking. Most studies on neural speech tracking report findings of frequencies below 7 Hz; we, therefore, analyzed frequencies between 2 and 7 Hz. We applied repeated measures ANOVAs for each frequency within the range (*ft_statfun_depsamplesFunivariate* in FieldTrip) to test modulations of neural measures across the different intelligibility levels. To control for multiple comparisons, a nonparametric cluster‐based permutation test was undertaken (Maris & Oostenveld, 2007). The test statistic was repeated 10,000 times on data shuffled across conditions and the largest statistical value of a cluster coherent in source space was kept in memory. The observed clusters were compared against the distribution obtained from the randomization procedure and were considered significant when their probability was below 5%. Effects were identified in source space. All virtual channels within the cluster and the corresponding individual coherence and power values were extracted and averaged. Post‐hoc paired samples *t‐*tests between conditions were corrected for multiple comparisons by using the FDR method (Benjamini & Hochberg, 1995) implemented in Pingouin (Vallat, 2018). Slopes of the change in coherence along with changes in intelligibility were compared across studies using a Mann–Whitney *U* test. For visualization, source localizations were averaged across the 2–7 Hz frequency bands and mapped onto inflated surfaces as implemented in FieldTrip.

### 5.8 Peak analysis

For further analysis of the coherence spectra in source space, we extracted the most prominent peaks in the low‐frequency range (2–7 Hz) across all virtual channels in which we observed a significant difference across vocoding levels (579 channels for Study#1 and 417 for Study#2; see Figure 2b,e). This was operationalized by using FOOOF (Donoghue et al., 2020) to flatten the coherence spectrum at each virtual channel and compute Gaussian model fits to extract peaks. For each subject, the average peak height, bandwidth, and center frequency of the extracted peaks (see Figure 3) were computed. Peaks were only considered if they exceeded a threshold relative to the aperiodic slope of 1.5 standard deviations (peak_threshold = 1.5). Bad model fits were dropped (one bad model fit in Study#2). If the residual model fits differed from the rest based on the *R* ^*2*^ (between the input spectrum and the full model fit), or error of the full model fit by more than 2.5 *SDs*, they were dropped. The most prominent peak in the range between 2 and 7 Hz was extracted per virtual channel. Peak and aperiodic parameters were then averaged across all virtual channels and further analyzed using repeated measures ANOVAs and dependent‐samples *t*‐tests (as implemented in Pingouin (Vallat, 2018)) for post‐hoc analysis (corrected for multiple comparisons using the FDR method (Benjamini & Hochberg, 1995)).

### 5.9 Analysis of modulation and syllable rate

We estimated the modulation and syllabic rate of all 12 audio files for each condition (Original, 7‐Channels, and 3‐Channels). Audio files were transformed to 6‐s duration segments (as in (Ding et al., 2017)) resulting in 386 audio segments per condition (Original, 7‐Channels, and 3‐Channels). The modulation rates for the three different levels of intelligibility were then extracted using custom matlab scripts taken from (Ding et al., 2017). The center frequency of each spectrum was further extracted by taking the global maximum value of each modulation spectrum. The realized syllable rate of the speaker was computed using Praat (de Jong & Wempe, 2009). The center frequencies of the three conditions and syllable rate were then compared using repeated measures ANOVAs and dependent‐samples *t*‐tests (as implemented in Pingouin (Vallat, 2018)) for post‐hoc analysis (corrected for multiple comparisons using the FDR method (Benjamini & Hochberg, 1995)). We further calculated the absolute difference between the different modulation/syllable rates and the center frequency of the low‐frequency coherence peaks. The corresponding absolute differences between syllable and modulation rates were then compared using a two‐way repeated measures ANOVA with the factors tracking (modulation/syllable rate) and vocoding (Original, 7‐Channels, and 3‐Channels). We further validated the resulting findings using a different analysis approach based on decoding. Here, we trained an ensemble (50 classifiers) of *k*‐nearest neighbor classifiers in a nested fivefold cross‐validation (Hosseini et al., 2020) to decode whether a given frequency in the presented stimulus material can be associated with either the modulation or the syllabic rate. We decided to use the *k*‐nearest neighbor classifiers as data had only a low number of features (i.e., one center frequency per audio segment); a classification problem usually solved well by a *k*‐nearest neighbor approach (Eisa et al., 2018). The repeated nested cross‐validation procedure was chosen to avoid overfitting of hyperparameters. Each external cross‐validation loop was embedded in a repeated stratified *k*‐folding procedure (*RepeatedStratifiedKFold;* 25 repetitions) the best number of neighbors was determined by searching the hyper‐parameter space for the best cross‐validation (CV) score of a kNN model using the implemented *GridSearchCV* function and by computing the area under the receiver operating characteristic curve (*roc‐auc*) as loss function. Confusion matrices were then computed on a separated test set (10% of all data) that was not part of the initial inner cross‐validation to avoid overfitting of hyperparameters. Confusion matrices of each inner loop were kept in memory and averaged across all repetitions (150 repetitions). The procedure was implemented using sci‐kit learn (Pedregosa et al., 2011) and custom written python scripts. The code used for the analysis can be found in the corresponding authors gitlab repository (see data & code availability). The trained classifiers were subsequently applied to the center frequencies from speech‐brain coherence (see Section 5.8: Peak analysis) to determine whether a frequency was rather related to the modulation or the syllabic rate. The corresponding probabilities were then compared using a two‐way repeated measures ANOVA with the factors tracking (modulation/syllable rate) and vocoding (Original, 7‐Channels, and 3‐Channels).

### 5.10 Modeling of subcortical auditory activity

In order to reconstruct auditory brainstem activity from the MEG data we applied a recently developed backward modeling approach (Schmidt et al., 2020) to the data obtained in Study#2. As planar gradiometers are less sensitive to sources below the cortical surface than magnetometers (Vrba & Robinson, 2002) only magnetometer data were included in this analysis. The backward models were trained independently for each subject using data obtained from a localizer run dedicated to elicit auditory brainstem activity (see (Schmidt et al., 2020) for a detailed account). In brief, we used the signal captured by the MEG sensors (during the first 10 ms) as regressors for a concurrent EEG recording of an auditory brainstem response (similar to the estimation of regression‐based ERPs (Smith & Kutas, 2015)). The corresponding weights (a time‐generalized representation of auditory brainstem activity) were then applied to the upsampled (10,000 Hz) single‐trial time series data from Study#2. Afterwards, the data were downsampled (100 Hz) and a frequency analysis was applied to the 4‐s segments of all three conditions (Original, 7‐Channels, and 3‐Channels) calculating multi‐taper frequency transformation (dpss taper: 0–25 Hz in 0.25 Hz steps, 4 Hz smoothing, no baseline correction) for the analyses of the coherence calculation between each virtual sensor and the acoustic speech envelope. Afterwards analysis steps that were performed for the previous analysis were repeated for the modeled activity (see statistics reported in source analysis and steps undertaken for peak and decoding analysis).

## AUTHOR CONTRIBUTIONS

**Fabian Schmidt:** Conceptualization; data curation; formal analysis; investigation; methodology; software; visualization; writing – original draft; writing – review and editing. **Ya‐Ping Chen:** Conceptualization; data curation; formal analysis; investigation; writing – review and editing. **Anne Keitel:** Conceptualization; supervision; writing – review and editing. **Sebastian Roesch:** Funding acquisition; supervision. **Ronny Hannemann:** Funding acquisition; supervision; writing – review and editing. **Maja Serman:** Funding acquisition; supervision. **Anne Hauswald:** Conceptualization; data curation; formal analysis; investigation; software; supervision; writing – review and editing. **Nathan Weisz:** Conceptualization; funding acquisition; methodology; project administration; supervision; writing – review and editing.

## CONFLICT OF INTEREST STATEMENT

The authors declare no competing financial interests.

## DATA/CODE AVAILABILITY STATEMENT

The data and code necessary for generating the figures and computing statistics will be shared in the corresponding authors gitlab repository (https://gitlab.com/schmidtfa). Access to raw data will be made available upon reasonable request.

## Supporting information


**Text S1.** The degree of the 1/f slope in a coherence spectrum is related to differences in power law noise between signals.
**Text S2.** Decreases in intelligibility can be associated with a lower offset and flatter slope of low frequency speech‐brain coherence.
**Text S3.** Aperiodic components explain most of the variance of low frequency speech‐brain coherence.
**Text S4.** Decoding center frequencies of speech tracking based on acoustic and linguistic rates.
**Figure S1.** The degree of the 1/f slope in a coherence spectrum is related to differences in power law noise between signals.
**Figure S2.** Decreases in intelligibility can be associated with a lower offset and flatter slope of low frequency speech‐brain coherence.
**Figure S3.** Aperiodic components explain most of the variance of low frequency speech‐ brain coherence.
**Figure S4.** Decoding center frequencies of speech tracking based on acoustic and linguistic rates.
**Figure S5.** Parametrized grand‐average speech‐brain coherence spectra.
**Table S1.** Differences across the extracted center frequencies and the syllable rate of the audio signal.
**Table S2.** Center Frequencies and syllable rate of the audio signal.

## Data Availability

The data and code necessary for generating the figures and computing statistics will be shared in the corresponding authors gitlab repository (https://gitlab.com/schmidtfa). Access to raw data will be made available upon reasonable request.
