# Supplementary material for: Neural speech tracking shifts from the syllabic to the modulation rate of speech as intelligibility decreases
Source: Psychophysiology. 2023 Jun 23;60(11):e14362. doi: 10.1111/psyp.14362 (PMC10909526; doi:10.1111/psyp.14362)
Supplement: Supplementary file 1 — Text S1. The degree of the 1/f slope in a coherence spectrum is related to differences in power law noise between signals. Text S2. Decreases in intelligibility can be associated with a lower offset and flatter slope of low frequency speech‐brain coherence. Text S3. Aperiodic components explain most of the variance of low frequency speech‐brain coherence. Text S4. Decoding center frequencies of speech tracking based on acoustic and linguistic rates. Figure S1. The degree of the 1/f slope in a coherence spectrum is related to differences in power law noise between signals. Figure S2. Decreases in intelligibility can be associated with a lower offset and flatter slope of low frequency speech‐brain coherence. Figure S3. Aperiodic components explain most of the variance of low frequency speech‐ brain coherence. Figure S4. Decoding center frequencies of speech tracking based on acoustic and linguistic rates. Figure S5. Parametrized grand‐average speech‐brain coherence spectra. Table S1. Differences across the extracted center frequencies and the syllable rate of the audio signal. Table S2. Center Frequencies and syllable rate of the audio signal. [file PSYP-60-e14362-s001.docx]

# SUPPLEMENTARY MATERIAL

## Text S1. The degree of the 1/f slope in a coherence spectrum is related to differences in power law noise between signals

In the current study we observed a 1/*f^x^* decay in speech-brain coherence. The presence of a 1/*f^x^* decay can also be found/seen in previous studies investigating low-frequency speech-brain coherence/correlation (e.g. see [(Ding et al., 2014; Gross et al., 2013; Hauswald et al., 2020)](https://www.zotero.org/google-docs/?VkWHt6)). However, the mechanism generating this 1/f^x^ in the recorded data remains unclear. Given that both spectral power in recordings of neural activity (Donoghue et al. 2020) and the envelope of speech (Poeppel & Assaneo, 2020) decay 1/f^x^ we hypothesized that potential differences in the signal-to-noise ratio between both signals may introduce a 1/f^x^ in the coherence spectrum. We expected that this difference arises either in the form of increased noise across all frequencies (reflecting a broadband difference in noise between both recordings) or in the 1/f slope between both signals (reflecting differences in 1/f^x^ noise in both recordings). To gain a mechanistic understanding of this process we simulated neural time series (Cole et al., 2019) with different noise characteristics and calculated coherence.

A “reference signal” - a 10min time series with a sampling rate of 1000Hz, an oscillation at 4Hz and a power law component (set to 1.5) - was generated using *neurodsp* [(Cole et al., 2019)](https://www.zotero.org/google-docs/?YP3FAz). Either white noise in different intensities or power law noise with different exponents was added to the “reference signal”. Afterwards the coherence between the “reference signal” and each altered version of the “reference signal” was calculated (see Fig. S1).

The results of this analysis indicate that when adding broadband noise and therefore artificially whitening the spectrum of the “reference signal” while keeping the y-intercept (offset) constant the slope in the coherence spectrum increases (see Fig. S1A). Furthermore, the slope of the coherence spectrum can also be changed by changing the 1/f exponent in the added noise. Notably, if the added noise has the same exponent as the reference signal the slope of the coherence spectrum is flat (see Fig. S1Bii yellow line outlined in black). This may also explain why a 1/f characteristic in coherence is not a common sight when computed between brain regions (where large differences in 1/f are uncommon). However, if the 1/f slope of the added signal is flatter than the 1/f of the reference signal the coherence decreases 1/f and if the component of the added signal exceeds the 1/f in the reference signal the coherence increases 1/f. Our results show that a 1/f decay in a coherence spectrum may unfold in at least two different ways related to differences in the signal-to-noise ratio between two signals. Importantly, the differences in signal-to-noise ratio in the simulated signals affected the slope of the coherence spectrum, but not the oscillation simulated at 4Hz. This highlights the importance of separating periodic from aperiodic signals when calculating the coherence between two signals that also have a different slope in their power spectra to better interpret narrowband differences in spectral coherence.


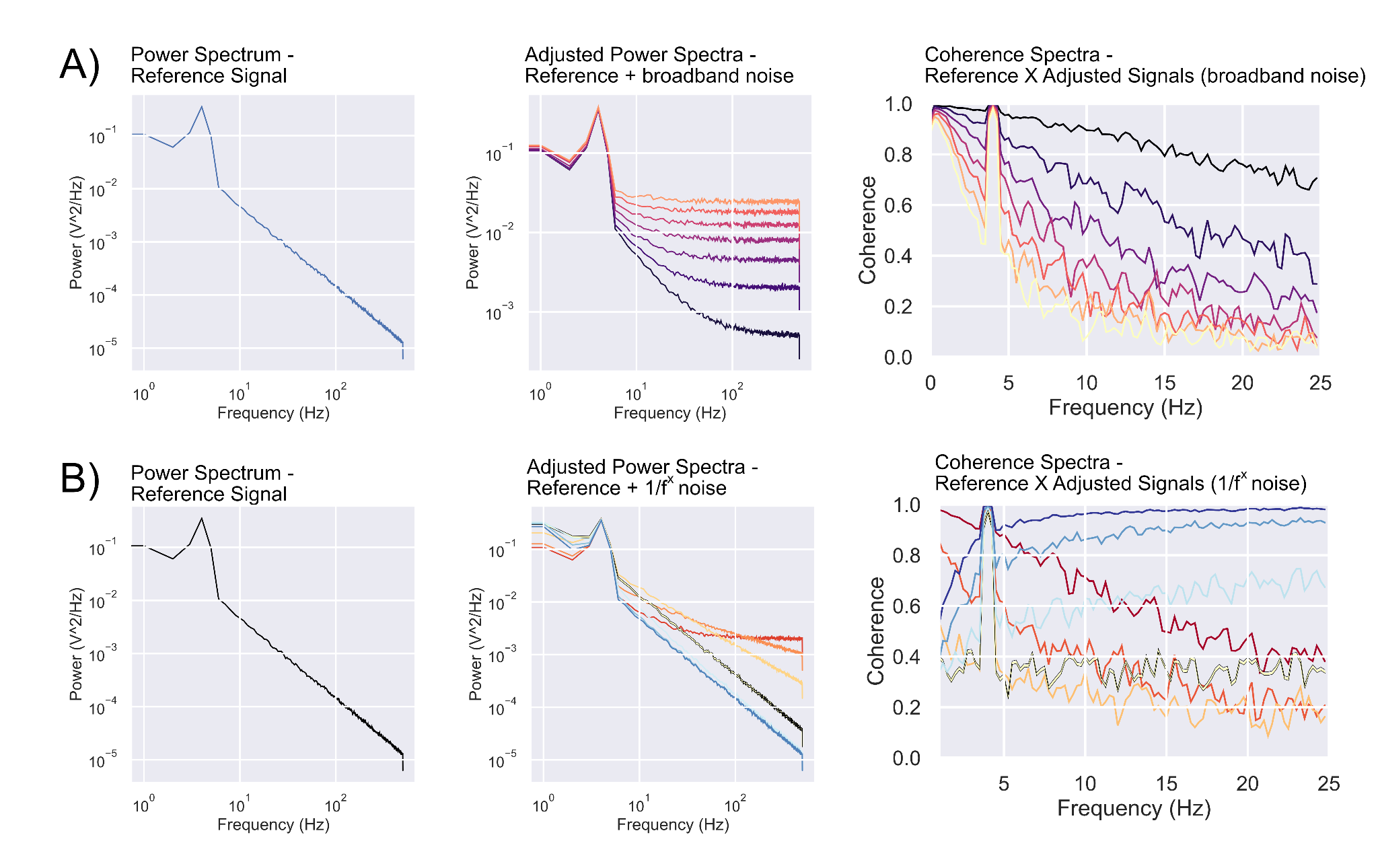


***Fig. S1*** **The degree of the 1/f slope in a coherence spectrum is related to differences in power law noise between signals.** ***(A)*** *The coherence between a “reference signal” and a version of the reference signal with added white noise was calculated. Increased broadband noise resulted in a stronger 1/f decay in the coherence spectrum.* ***(B)*** *Coherence calculated between a “reference signal” and differently colored noise. In the case that the exponent of the added 1/f noise exceeds the exponent of the reference signal coherence increases 1/f, if the exponent is lower than in the reference signal coherence decreases 1/f and if the exponent is exactly the same as in the reference signal no 1/f decay can be noted.*

##

## Text S2. Decreases in intelligibility can be associated with a lower offset and flatter slope of low frequency speech-brain coherence

In this study, we mainly focused on investigating the periodic components underlying speech-brain coherence. However, when separating the periodic from the aperiodic components we also noticed that exponent and offset of speech-brain coherence were modulated by intelligibility (see Fig. S2). We further investigated this using a repeated-measure ANOVA. This analysis showed that in both studies the exponent (Study#1, *F*(2, 54) = 45.898, *p_ggeisser_* = 2.329e^-10^, *η_p_^2^* = 0.630; Study#2, *F*(2, 50) = 18.409, *p* = 1e^-06^, *η_p_^2^* = 0.424) and the offset (Study#1, *F*(2, 54) = 51.793, *p* = 2.765e^-13^, *η_p_^2^* = 0.657; Study#2, *F*(2, 50) = 35.804, *p* = 2.239e^-10^, *η_p_^2^* = 0.589) differed significantly across the three vocoding conditions. Comparing the different vocoding levels with each other showed a higher coherence offset for unvocoded stimuli than for stimuli vocoded with 7-Channels (Study#1, *t*(27) = 3.505, *p_fdr_* = 0.0016, *d* = 0.744; Study#2, *t*(25) = 2.214, *p_fdr_* = 0.0361, *d* = 0.466) and with 3-Channels (Study#1, *t*(27) = 8.289, *p_fdr_* = 1.598e^-08^, *d* = 2.156; Study#2, *t*(25) = 8.581, *p_fdr_* = 1.919e^-08^, *d* = 2.031). Additionally stimuli vocoded with 7-Channels had a higher offset than stimuli vocoded with 3-Channels (Study#1, *t*(27) = 7.559, *p_fdr_* = 5.912e^-08^, *d* = 1.486; Study#2, *t*(25) = 5.676, *p_fdr_* = 6.558e^-06^, *d* = 1.277).

Furthermore, the exponent of the slope of the coherence spectra flattened with intelligibility. Unvocoded stimuli elicited a steeper slope in the coherence spectra than stimuli vocoded with 7-Channels (in Study#1; *t*(27) = 3.841, *p_fdr_* = 0.0007, *d* = 0.936, but not in Study#2 (*t*(25) = 1.268, *p_fdr_* = 0.216, *d* = 0.259) and stimuli vocoded with 3-Channels (Study#1, *t*(27) = 7.961, *p_fdr_* = 4.431e^-08^, *d* = 2.304; Study#2, *t*(25) = 6.171, *p_fdr_* = 6e^-06^, *d* = 1.488). Additionally stimuli vocoded with 7-Channels elicited a steeper slope than stimuli vocoded with 3-Channels (Study#1, *t*(27) = 7.528, *p_fdr_* = 6.377e^-08^, *d* = 1.246; Study#2, *t*(25) = 4.047, *p_fdr_* = 0.0007, *d* = 0.993).

In sum, we show that as intelligibility decreases the offset of speech-brain coherence decreases and the slope of the coherence spectra becomes flatter. These differences in the aperiodic parameters may also partly account for band-limited differences in low frequency speech-brain coherence that are commonly related and also interpreted as differences in neural speech tracking. Yet, the separation of broadband coherence in periodic and aperiodic components illustrates that analyzing coherence in a band-limited range, even though more or less explicitly assumed, may not reflect neural tracking of sound or linguistic information in the relevant frequency range. But to what extent are these parameters related to the band-limited differences in low frequency speech tracking that we measured (see Fig. 2)?


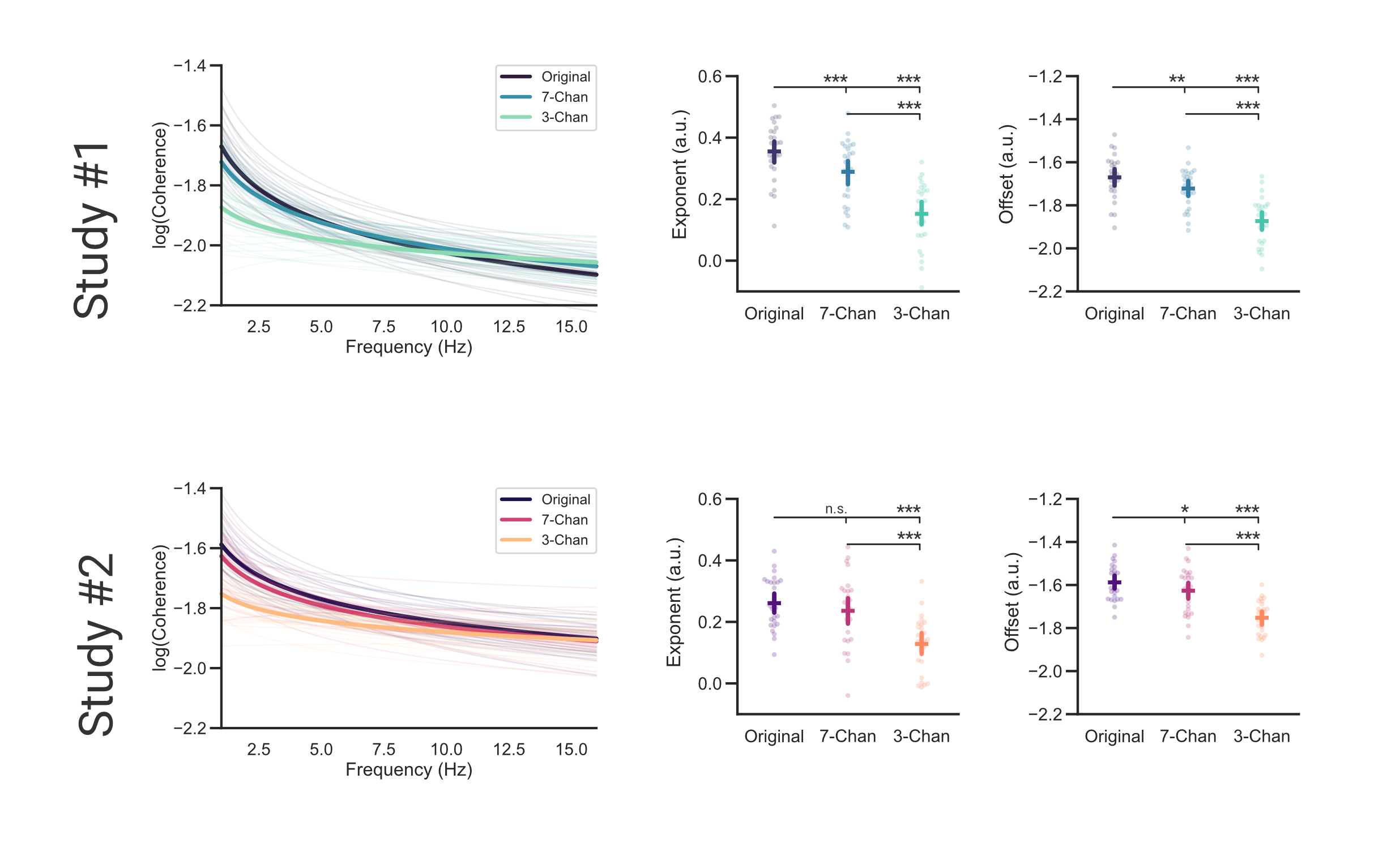


***Fig. S2*** ***Decreases in intelligibility can be associated with a lower offset and flatter slope of low frequency speech-brain coherence.*** *The averaged exponents and offsets extracted from the coherence spectra for each subject were compared across three conditions (Original, 7-Chan & 3-Chan). Bars represent 95% confidence intervals, p_fdr_ < 0.05*, p_fdr_ < 0.01**, p_fdr_ < 0.001****

## Text S3. Aperiodic components explain most of the variance of low frequency speech-brain coherence

In order to better understand the extent to which aperiodic components explain the effect we showed for low frequency speech-brain coherence (see Fig. 2) we calculated a repeated measures correlation [(Bakdash & Marusich, 2017)](https://www.zotero.org/google-docs/?rsETvp) between the averaged coherence in the low frequency range (2-7Hz) and the periodic and aperiodic components in the same frequency range across all vocoding levels (Original, 7-Channels, 3-Channels). The results of this analysis showed that low-frequency speech-brain coherence showed overall higher correlation coefficients with the underlying aperiodic components offset (Study#1, *r* (55) *=* 0.91, *p* = 8.476e^-23^, *r^2^* = 0.83; Study#2, *r* (51) *=* 0.6, *p* = 2e^-06^, *r^2^* = 0.36) and exponent (Study#1, *r* (55) *=* 0.82, *p* = 8.726e^-15^, *r^2^* = 0.67; Study#2, *r* (51) *=* 0.57, *p* = 1e^-05^, *r^2^* = 0.32) compared to the periodic components center frequency (Study#1, *r* (55) *=* -0.56, *p* = 7e^-06^, *r^2^* = 0.31; Study#2, *r* (51) *=* -0.22, *p* = 0.121, *r^2^* = 0.05), bandwidth (Study#1, *r* (55) *=* 0.7, *p* = 1.948e^-09^, *r^2^* = 0.48; Study#2, *r* (51) *=* 0.41, *p* = 0.002, *r^2^* = 0.17) and the relative magnitude of the coherence peak (Study#1, *r* (55) *=* 0.33, *p* = 0.011, *r^2^* = 0.11; Study#2, *r* (51) *=* 0.00, *p* = 0.998, *r^2^* = 0.00).

In sum, these findings illustrate the importance of parametrizing speech-brain coherence spectra to remove the influence of aperiodic components from the periodic components to better understand the parameters that actually reflect neural speech tracking.

***
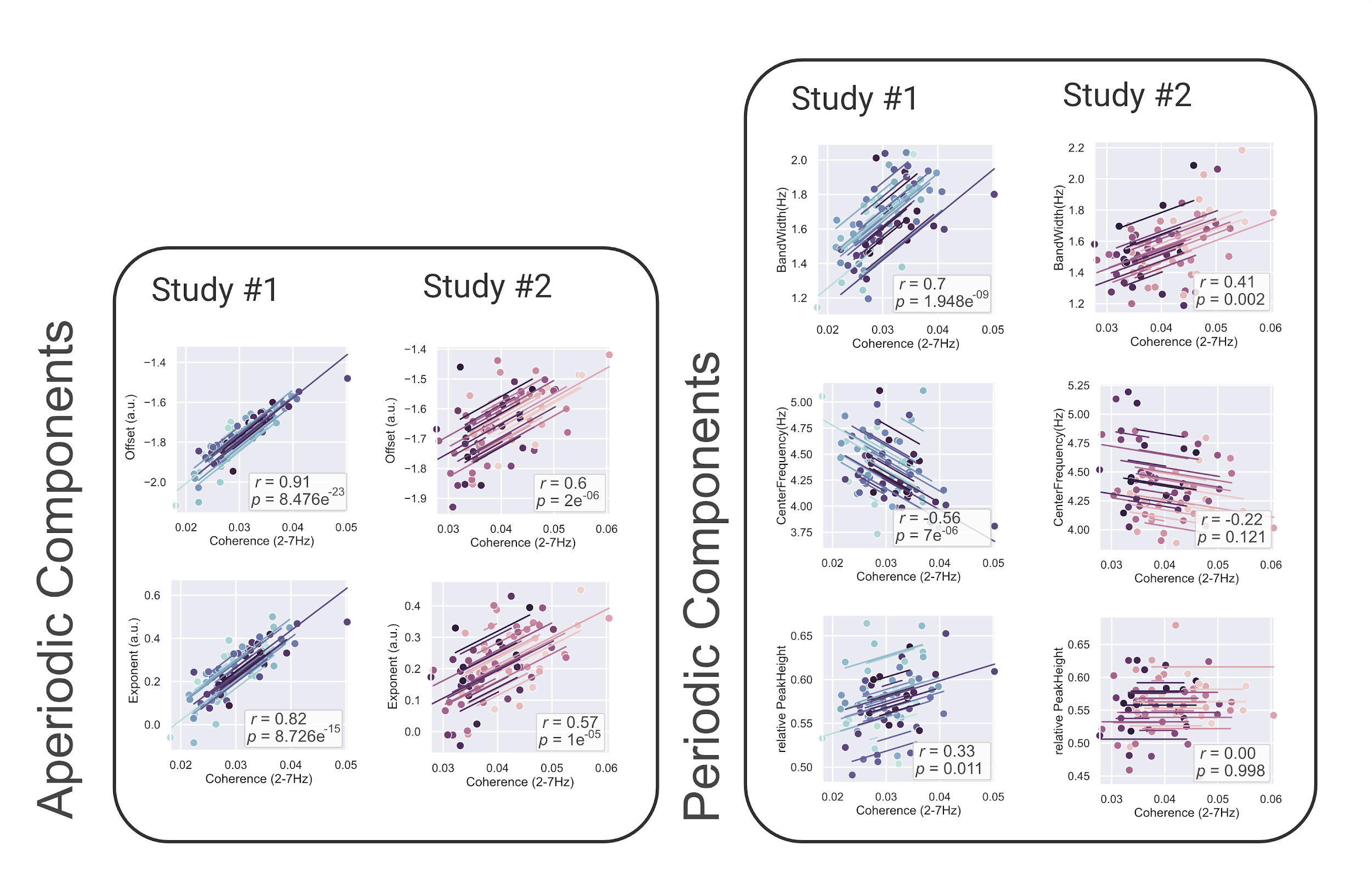
***

***Fig. S3 Aperiodic components explain most of the variance of low frequency speech- brain coherence****. Using a repeated measures correlation analysis we show that the aperiodic components (offset, exponent) of speech-brain coherence were related stronger to the band-limited (2-7Hz) averaged speech-brain coherence than the periodic components (center frequency, relative magnitude, bandwidth). This highlights the importance of parametrizing speech-brain coherence to better understand neural speech tracking.*

##

## Text S4: Decoding center frequencies of speech tracking based on acoustic and linguistic rates

In order to collect additional evidence, whether changes in center frequencies of speech-brain coherence can be conceptualized as a shift between the tracking of acoustic and linguistic information, we trained and tested an ensemble of *k*-nearest neighbor classifiers. First, we performed a nested 5-fold cross-validation (see Method for a detailed account) to differentiate between the center frequencies of the modulation spectra for the three conditions (Original, 7-Channels, 3-Channels) and the realized syllable rate of the speaker (see Fig. 5A). Classifiers were trained separately for each speech condition (Original, 7-Channels, 3-Channels) to decode between the syllabic and the respective modulation rates. The training and validation was performed separately to account for a potential bias introduced by the different center frequencies of the modulation rates across speech conditions (see Fig. 5A). The results of the nested cross-validation procedure (Fig. S4A) show that the classifiers can predict with a high accuracy (>90% in all conditions) whether a given frequency in hertz can be related either to the modulation or realized syllable rate of the speaker in all conditions. We then used the weights of these classifiers to predict whether the extracted center frequencies of speech brain coherence were related more closely to the realized syllable rate or the modulation rate of our speaker. This analysis showed that in the unaltered clear speech condition, neural speech tracking was closely related to the syllable rate. However, as intelligibility decreases the probability that the classifiers predict that a given center frequency is related rather to the modulation as opposed to the syllabic rate increases. The results of a two-way repeated measures ANOVA revealed that there was a significant main effect for the factors tracking (Modulation/Syllable rate) in both studies (Study#1 *F*(1, 27) = 16.175, *p_ggeisser_* = 0.0004, *η_p_^2^* = 0.375; Study#2 *F*(1, 25) = 18.999, *p_ggeisser_* = 0.0002, *η_p_^2^* = 0.432). The probability that neural speech tracking is reflective of the syllable rate (linguistic component) was overall higher than the tracking of the modulation rate (acoustic component). There was no significant main effect of Vocoding (Original, 7-Channels, 3-Channels; Study#1 *F*(2, 54) = 0, *p* = 1; Study#2 *F*(2, 48) = 0, *p* = 1) this is intuitive as the overall probability in each condition is 0.5 when ignoring the factor tracking (Modulation/Syllable rate). However, there was a significant interaction effect for the factors tracking (Modulation/Syllable rate) and vocoding (Original, 7-Channels, 3-Channels) across both Studies (Study#1 *F*(2, 54) = 47.340, *p_ggeisser_* = 1.387e^-11^, *η_p_^2^* = 0.637; Study#2 *F*(2, 50) = 10.235, *p_ggeisser_* = 0.0006, *η_p_^2^* = 0.29). These results provide additional evidence to support the idea that while speech intelligibility decreases and less linguistically meaningful information is present, neural speech tracking starts to drift away from the syllabic rate towards the modulation rate of speech. Interestingly, a similar pattern was observable for modeled subcortical processing stages. However, contrary to the cortical recordings, the applied decoding analysis showed that the center frequencies of the speech-brain coherence peaks (reflecting neural speech tracking) across different levels of intelligibility at subcortical processing stages was predominantly related to the modulation rate of speech opposed to the syllabic rate. This shows that although tracking at a subcortical level is overall higher for the low-level acoustic envelope modulation, intelligibility also influences these hierarchically early responses (see Fig. S4C | Study#2 - Subcortical).


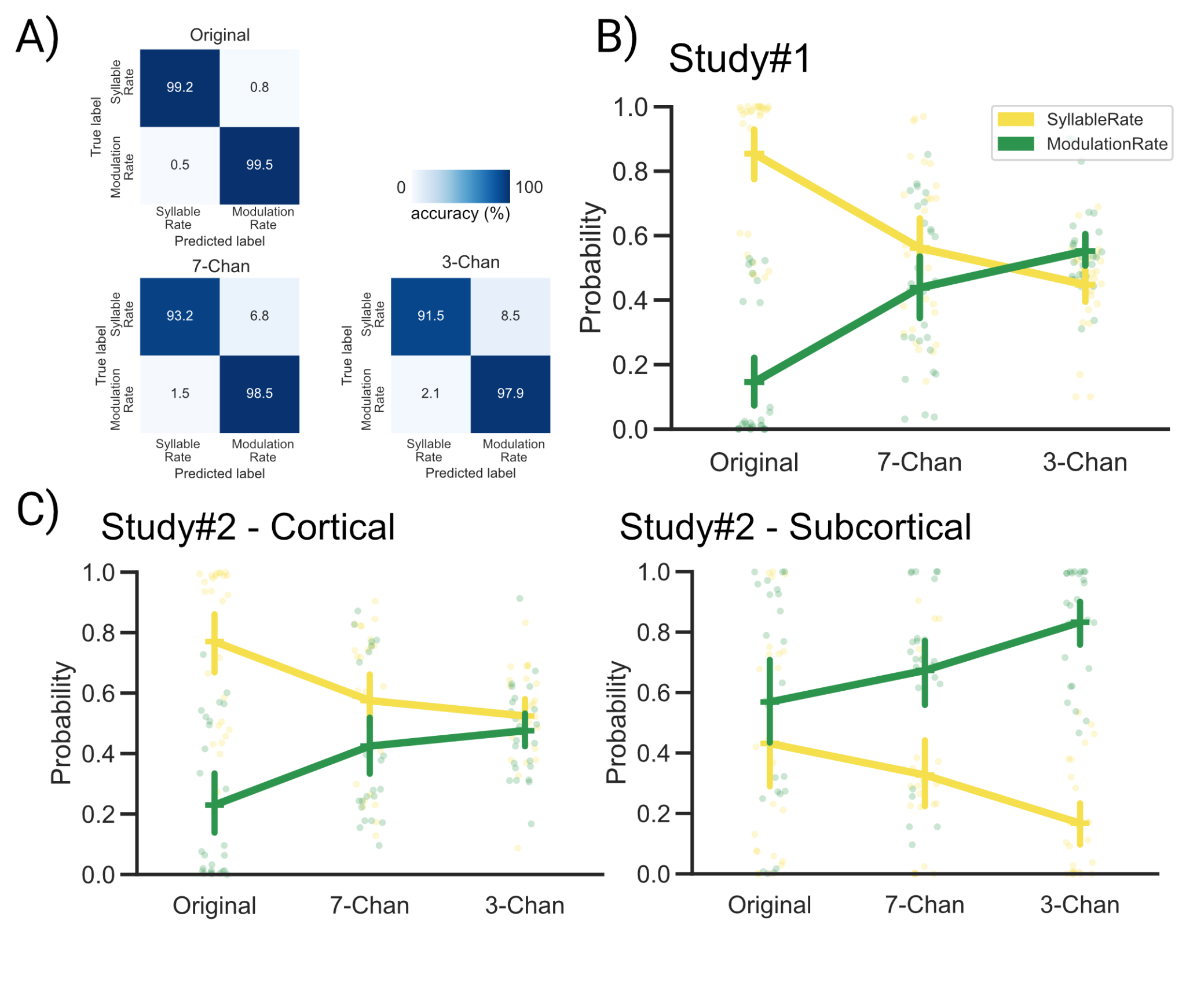
***Fig. S4 Decoding center frequencies of speech tracking based on acoustic and linguistic rates*** *(****A****) An ensemble of k-nearest neighbors classifiers were trained in a nested cross validation scheme to decode the modulation rate vs. the syllabic rate. (****B/C****) Classifiers applied to the center frequencies of the coherence spectra to decode whether the tracking was either related to the tracking of the syllable or the modulation rate of speech. Bars represent 95% confidence intervals, p_fdr_ < 0.05*, p_fdr_ < 0.01**, p_fdr_ < 0.001****

##

##

## Table S1. Differences across the extracted center frequencies and the syllable rate of the audio signal. Post-Hoc analysis was performed using fdr_bh_ corrected paired samples wilcoxon signed-rank tests implemented in pingouin [(Vallat, 2018)](https://www.zotero.org/google-docs/?fvjSiF)

| A | B | W-val | *p*_fdr_ | Cohen’s *d* |
| --- | --- | --- | --- | --- |
| CF 3-Chan | CF 7-Chan | 2730.5 | 8.847e^-12^ | -0.345 |
| CF 3-Chan | CF Original | 3688.5 | 5.478e^-26^ | -0.842 |
| CF 3-Chan | SyllableRate | 2642.5 | 5.271e^-52^ | 1.640 |
| CF 7-Chan | CF Original | 9390.5 | 1.716e^-10^ | -0.433 |
| CF 7-Chan | SyllableRate | 1591.0 | 1.022e^-55^ | 1.892 |
| CF Original | SyllableRate | 177.5 | 1.705e^-60^ | 2.747 |

## Table S2. Center Frequencies and syllable rate of the audio signal.

| Names | Mean | Standard Deviation |
| --- | --- | --- |
| CF Original | 5.95 Hz | 0.93 Hz |
| CF 7-Chan | 5.51 Hz | 1.1 Hz |
| CF 3-Chan | 5.15 Hz | 0.96 Hz |
| SyllableRate | 3.88 Hz | 0.52 Hz |

##

## Figure S5: Parametrized grand-average speech-brain coherence spectra


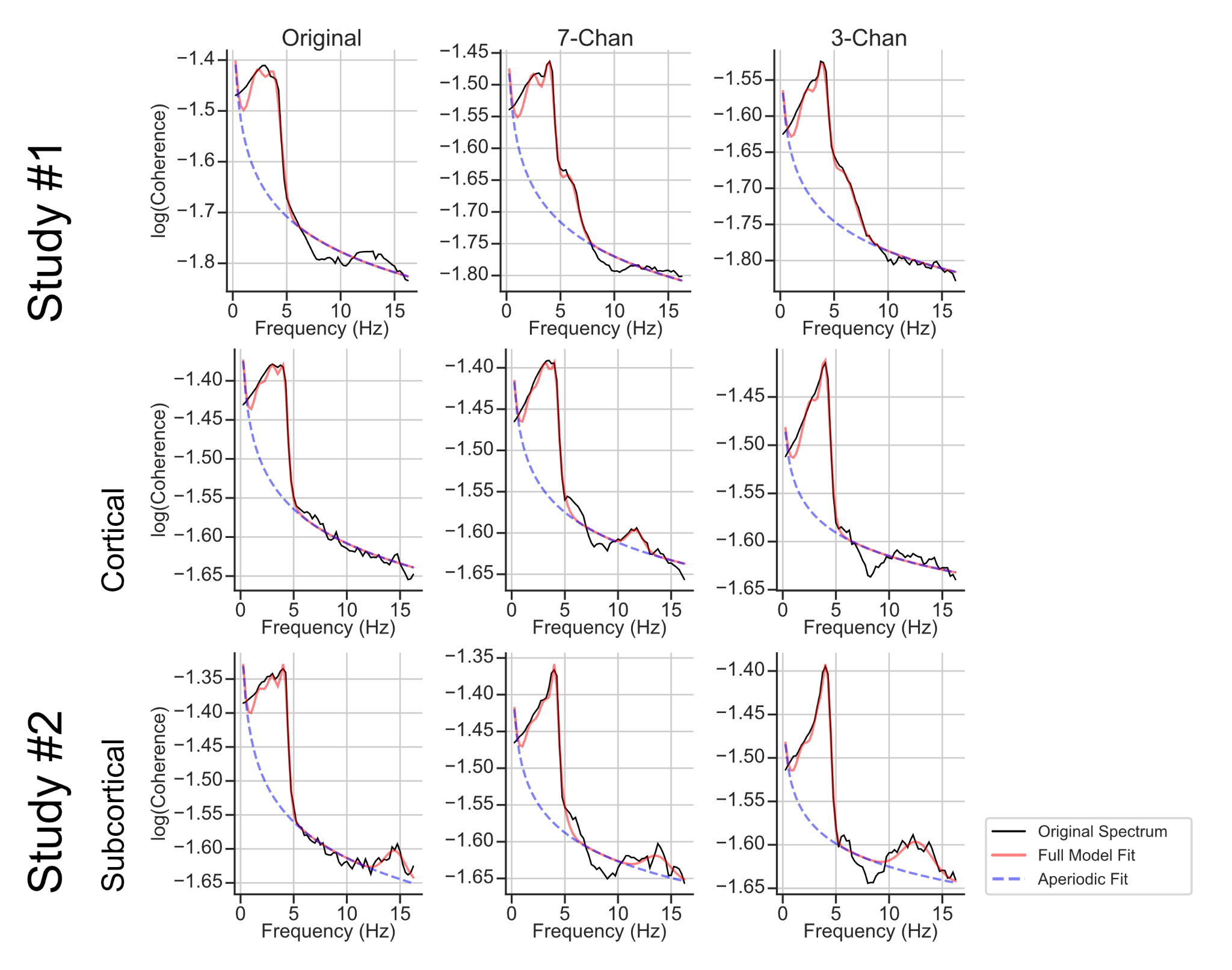


***Fig. S5:* Parametrized grand-average speech-brain coherence spectra. *(A)*** *shows the parametrized coherence spectra in Study#1 in the top-row.* ***(B)*** *shows the spectra for Study#2 separated in “cortical” (upper row) and “subcortical” speech-brain coherence.*
